# Supplementary material for: The IL6/JAK/STAT3 signaling axis is a therapeutic vulnerability in SMARCB1-deficient bladder cancer
Source: Nat Commun. 2024 Feb 14;15:1373. doi: 10.1038/s41467-024-45132-2 (PMC10867091; doi:10.1038/s41467-024-45132-2)
Supplement: Supplementary file 1 — Supplementary Information [file 41467_2024_45132_MOESM1_ESM.pdf]

Supplementary Information

The IL6/JAK/STAT3 signaling axis is a therapeutic vulnerability in SMARCB1-deficient bladder cancer

Supplementary Figures 1 – 14

Supplementary Tables 1 – 6

ARID1A

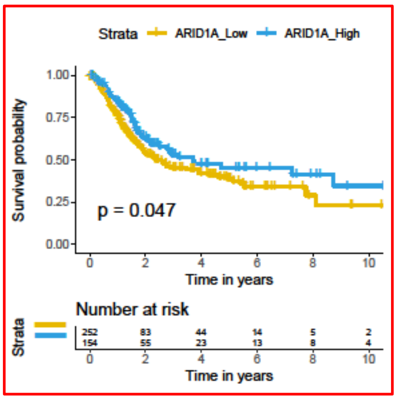

SMARCD1

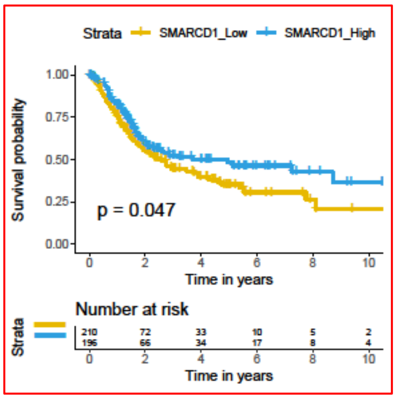

SMARCC1

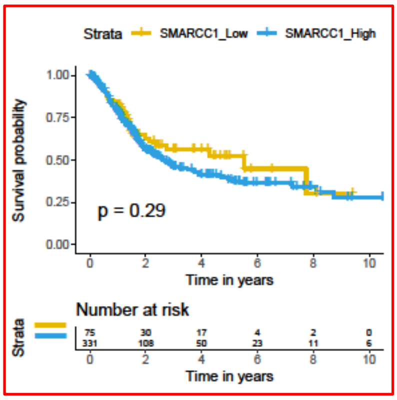

SMARCC2

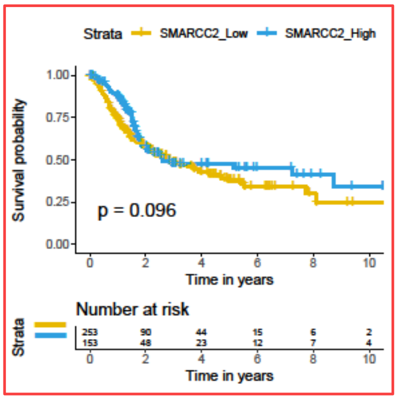

ARID1B

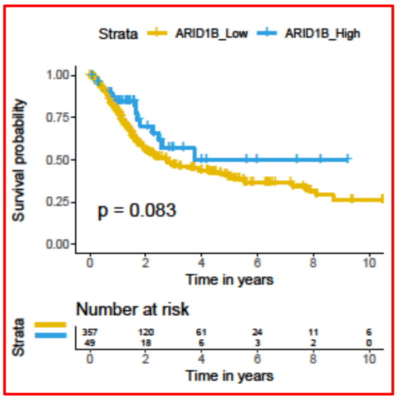

ARID2

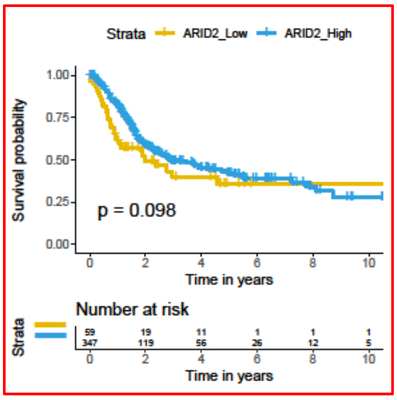

PBRM1

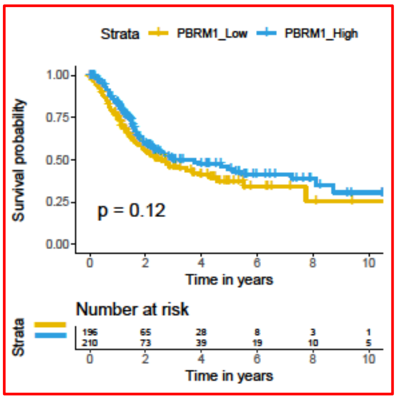

BRD7

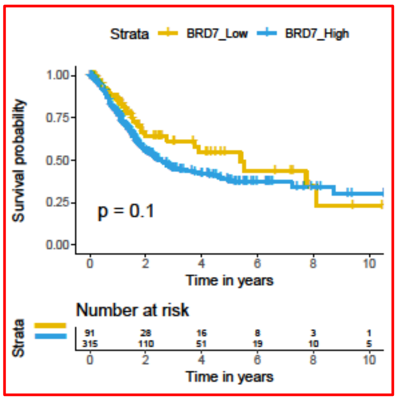

SMARCA4

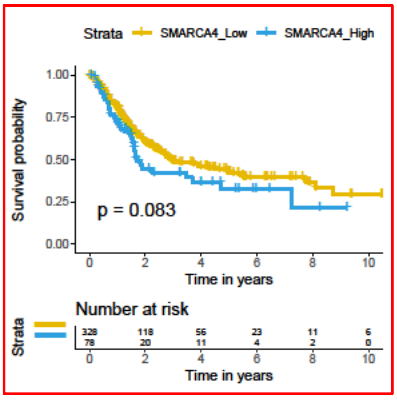

SMARCD2

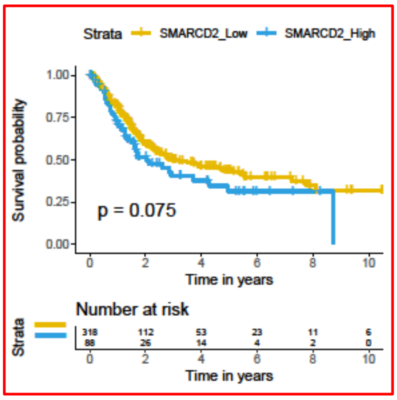

SMARCD3

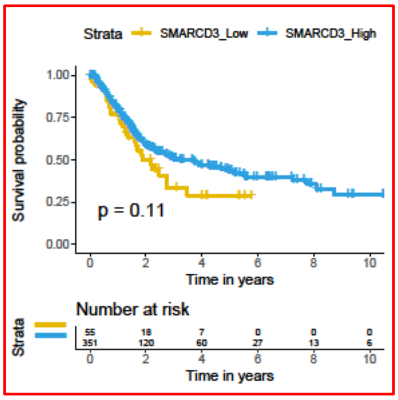

Supplementary Figure 1.

Supplementary Figure 1. Survival analysis of patients with BLCA based on ARID1A, SMARCD1, SMARCC1, SMARCC2, ARID1B, ARID2, PBRM1, BRD7, SMARCA4, SMARCD2 and SMARCD3 mRNA expression levels in TCGA-BLCA. Kaplan-Meier plots were generated for the TCGA-BLCA cohort, categorized by either low or high expression using maximally selected rank statistics. Low expression of ARID1A [low n=252; high n=154; log-rank test (p=0.047; two-sided)] and SMARCD1 [low n=210; high n=196; log-rank test (p=0.047; two-sided)] were associated with poor survival in BLCA based on maximally selected rank statistics. Low expression of SMARCC1 [low n=75; high n=331; log-rank test (p=0.29; two-sided)], SMARCC2 [low n=253; high n=153; log-rank test (p=0.096; two-sided)], ARID1B [low n=357; high n=49; log-rank test(p=0.083; two-sided)], ARID2 [low n=59; high n=347; log-rank test(p=0.098; two-sided)], PBRM1 [low n=196; high n=210; log-rank test (p=0.12; two-sided)], BRD7 [low n=91; high n=315; log-rank test(p=0.1; two-sided)], SMARCA4 [low n=328; high n=78; log-rank test (p=0.083; two-sided)], SMARCD2 [low n=318; high n=88; log-rank test (p=0.075; two-sided)] and SMARCD3 [low n=55; high n=351; log-rank test (p=0.11; two-sided)] did not show significance difference with patient survival.

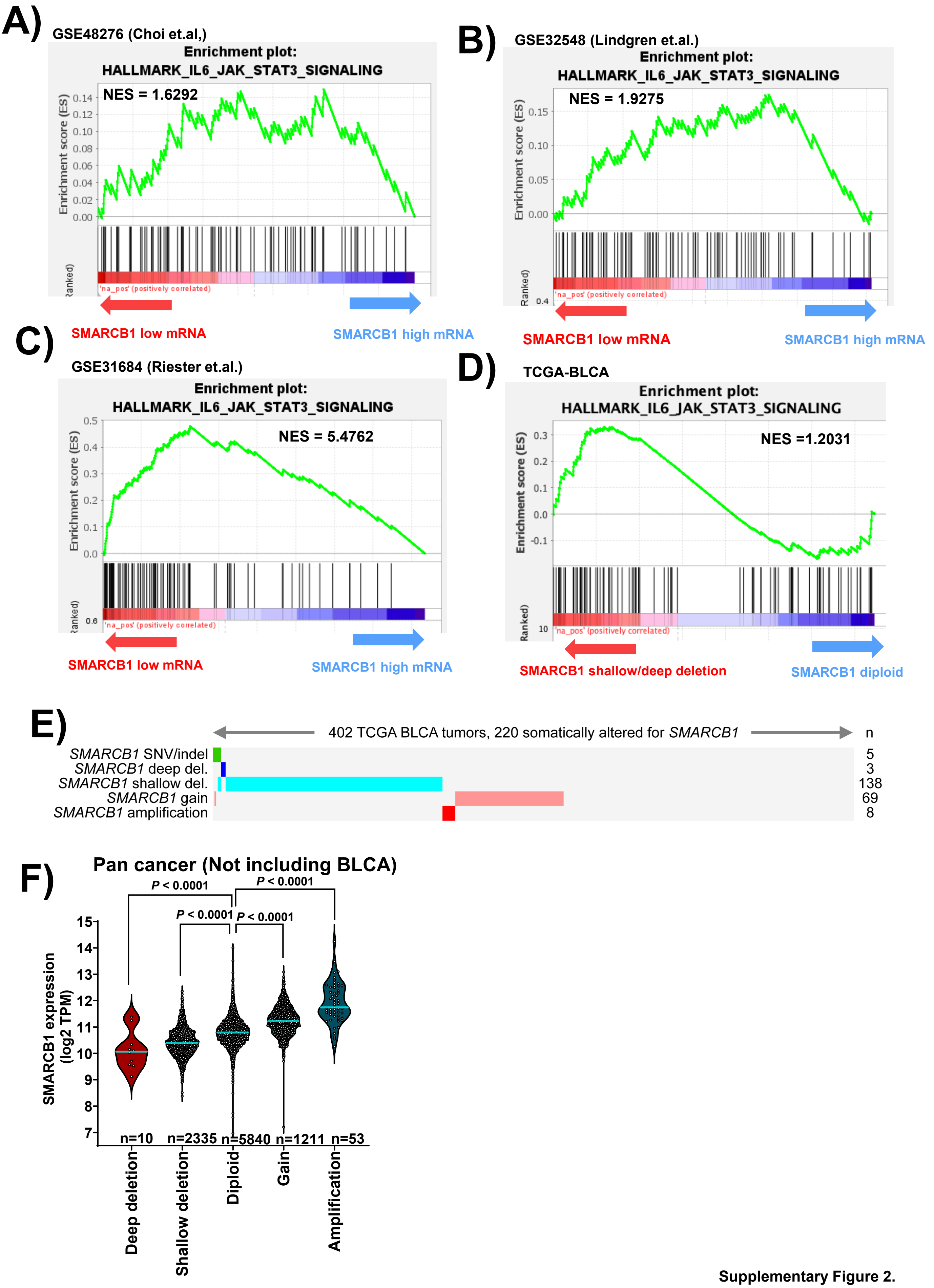

Supplementary Figure 2.

Supplementary Figure 2: Low SMARCB1 is associated with positive enrichment for STAT3 signaling. A-C) Gene set enrichment analysis (GSEA) of genes in the HALLMARK IL6/JAK/STAT3 signaling pathway in SMARCB1 low compared to SMARCB1 high patients in different BLCA cohorts [GSE48276 (Choi; NES=1.6292; p = 0.031), GSE32548 (Lindgren; NES=1.9275; p=0.012) and GSE31684 (Riester NES=5.4762; p < 0.0001)]. Genes in each dataset were ranked from low to high pearson's correlation with SMARCB1 expression (using log2- transformed expression values). **D)** GSEA of genes in the HALLMARK IL6/JAK/STAT3 signaling pathway comparing SMARCB1 deletion (deep/shallow; n=141) to SMARCB1 diploid (n=184) from TCGA-BLCA patients (FDR<0.25; NES=1.2031; p = 0.106). **E)** Oncoprint image of SMARCB1 in BLCA patients from TCGA cohort (n=402; only 5 patient have been identified with SMARCB1 indels). 4 patients do not have copy number alterations in the TCGA cohort and were not represented. **F)** Frequency of SMARCB1 alterations in pan cancer (excluding BLCA) from TCGA database, deep deletion (n=10); shallow deletion (n=2335), diploid (n=5840), gain (n=1211); amplification (n=53) (367 patients' copy number alteration (CNV) data was unavailable). Violin plots represent the expression levels of SMARCB1 mRNA expression with respect to copy number alterations of SMARCB1 in the pan cancer TCGA patient cohort [Data are represented as mean ± standard deviation (SD)]. For panel **F**, *P* values were determined by unpaired two-tailed Student's *t* test.

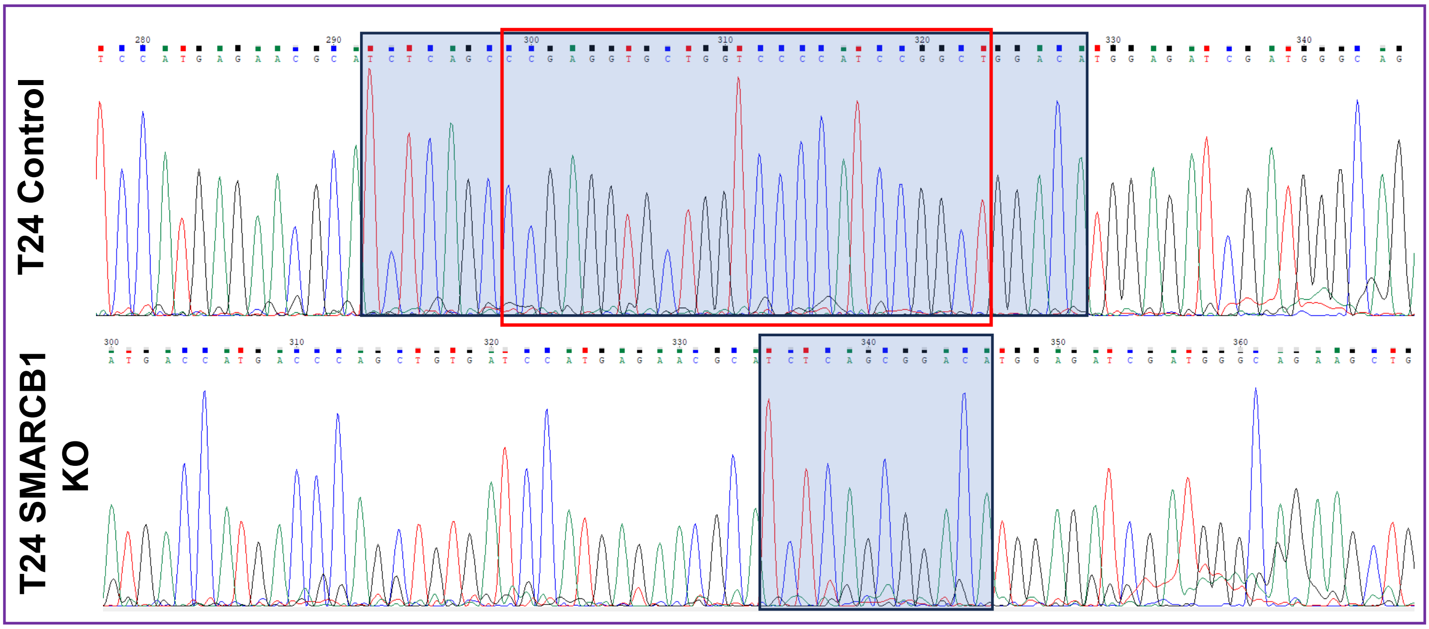

Supplementary Figure 3: Genomic DNA sequencing results of T24 Control and T24 SMARCB1 KO BLCA cells. Chromatograph shows the absence of sequence in SMARCB1 KO (clone 16) compared to control (Indicated by red box).

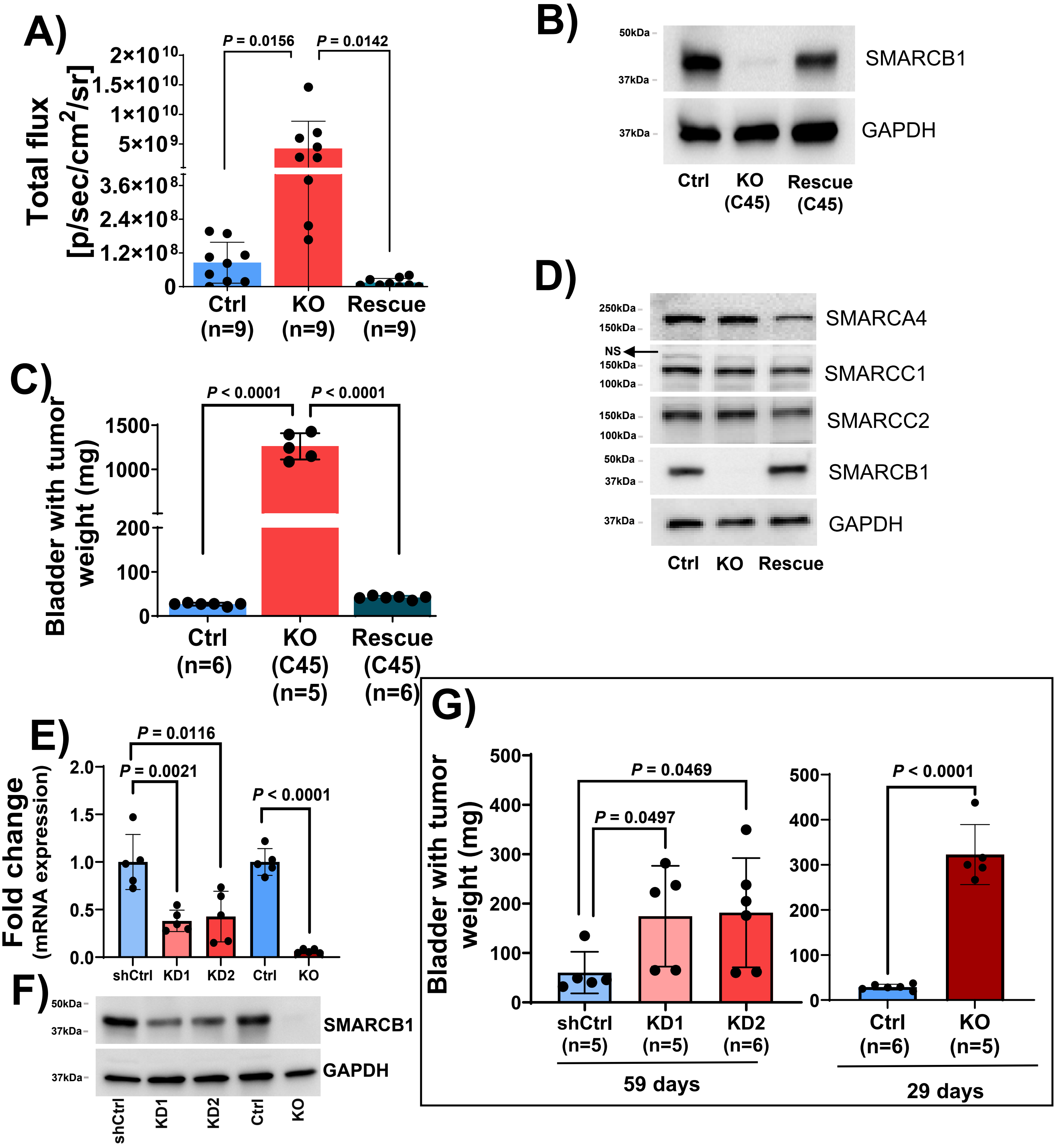

Supplementary Figure 4.

Supplementary Figure 4. SMARCB1 loss in BLCA promotes orthotopic tumor growth. A) The BLI signals of mice bearing T24 control (n=9). SMARCB1 KO (from clone 16) (n=9), and SMARCB1 rescue (n=9) cells were quantified on day 15 [Data are represented as mean ± standard deviation (SD)]. **B)** Validation of SMARCB1 loss (by CRISPR knockout (KO)- clone 45) and rescue (ectopic expression of SMARCB1 using KO –clone 45 cells) by immunoblot analysis using T24 bladder cancer cell lines. GAPDH was used as loading control. **C)** Weight of orthotopic bladders harboring tumors (day 16) from T24 control (n=6), SMARCB1 KO C45 (n=5), and SMARCB1 rescue C45 (n=6) [Data are represented as mean ± standard deviation (SD)]. **D)** Immunoblot analysis showing the expression levels of SMARCA4, SMARCC1, SMARCC2 and SMARCB1 (the core components of SWI/SNF complex) in T24 control, SMARCB1 KO (clone16) and SMARCB1 rescue cell lines. GAPDH was used as loading control (NS represents nonspecific bands). **E)** Relative mRNA fold change levels of SMARCB1 in T24 shCtrl cells (n=5 biological replicates each one analyzed under three technical replicates), SMARCB1 KD cells (Two independent shRNAs; five biological replicates each one analyzed under three technical replicates), T24 Ctrl cells (n=5 biological replicates each one analyzed under three technical replicates) and KO cell lines (n=5 biological replicates each one analyzed under three technical replicates) [Data are represented as mean ± standard deviation (SD)]. **F)** Immunoblot analysis showing the confirmation of SMARCB1 knockdown (Two independent shRNAs) and SMARCB1 knockout in T24 cell lines. **G)** Weight of bladders harboring orthotopic tumors from T24 shCtrl (n=5), SMARCB1 KD1 (n=5) and SMARCB1 KD2 (n=6) (End point on day 59); T24 control (n=6) and T24 SMARCB1 KO (n=5) (End point on day 29). [Data are represented as mean ± standard deviation (SD)]. For panels **A, C, E, G**, *P* values were determined by unpaired two-tailed Student's *t* test. Source data are provided as a Source Data file.

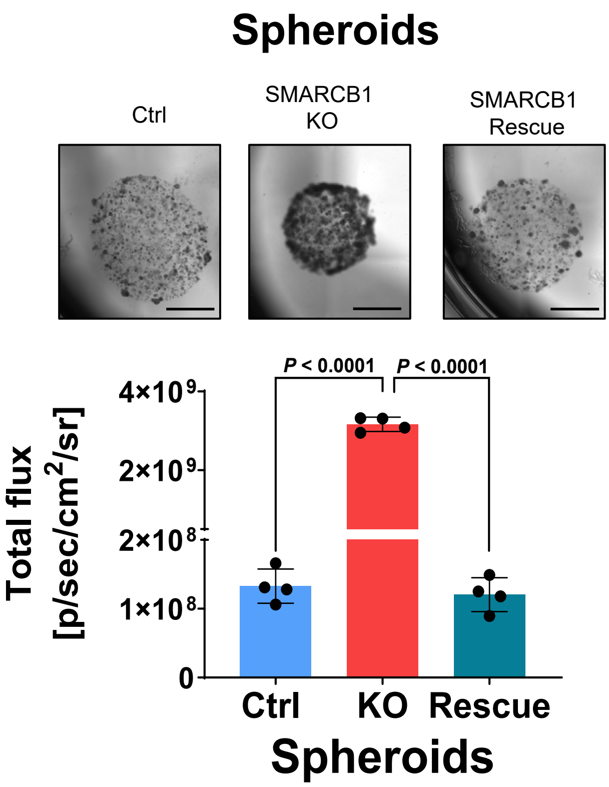

Supplementary Figure 5. SMARCB1 KO promotes BLCA spheroid growth *in vitro*. Images represent the spheroids from ctrl, SMARCB1 KO and rescue. Images were captured at 6 different focal points and stitched together by Biotek Gen5 software. Spheroid assay showing increased spheroid growth in SMARCB1 KO (clone 16) settings compared to ctrl and SMARCB1 rescue cell lines (n=4 replicates). Scale bar represents 2000μm. Scatter plot represents the quantification of BLI signal (total flux) in the spheroids on day 8. [Data are represented as mean ± standard deviation (SD)]. *P* values were determined by unpaired two-tailed Student's *t* test. Source data are provided as a Source Data file.

A)

Group	Lungs	Liver	Stomach/ Intestines	Kidneys
T24 Control	0/9	0/9	0/9	0/9
T24 SMARCB1 KO (C16)	7/7	7/7	7/7	3/7
T24 SMARCB1 rescue (C16)	0/9	0/9	0/9	0/9

B)

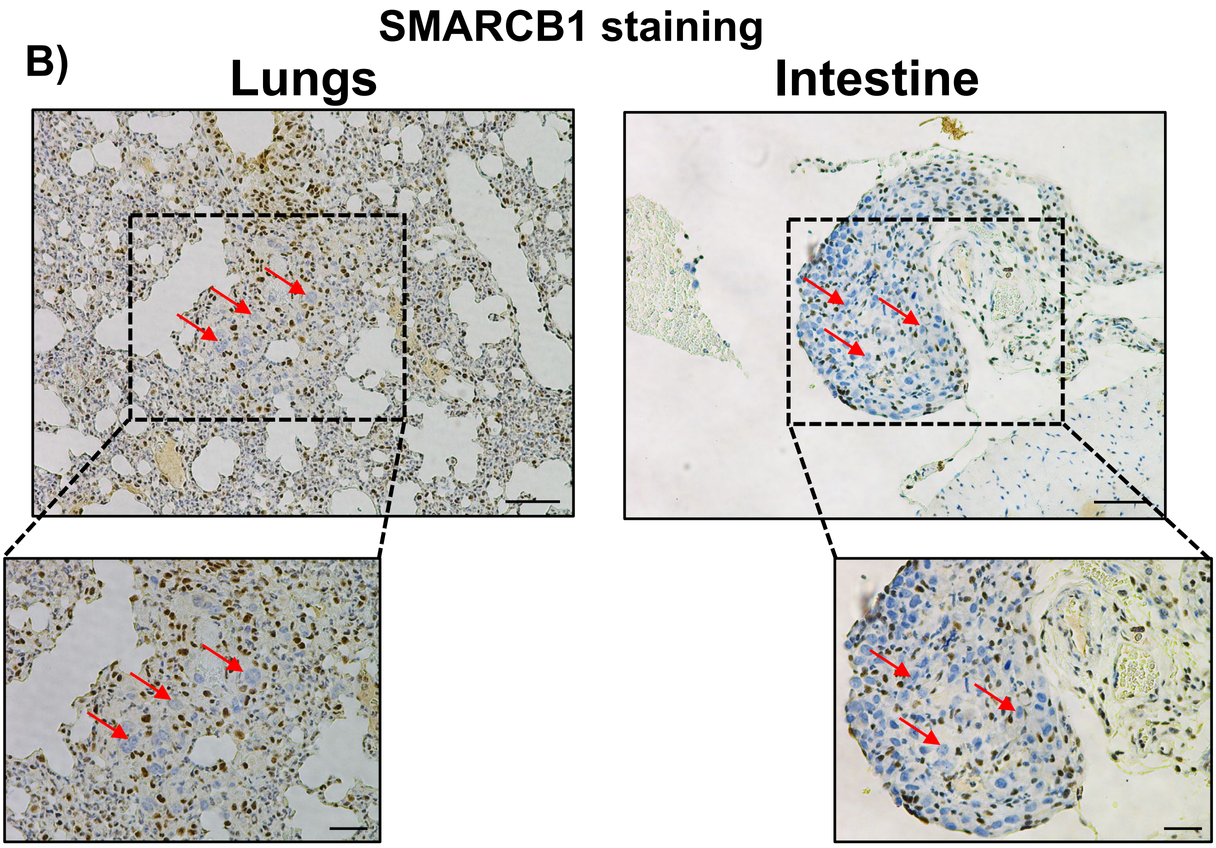

Supplementary Figure 6. Analysis of SMARCB1 KO induced metastatic phenotype in T24 orthotopic xenograft model. A) Table represents the number of ex vivo lungs, liver, kidney and stomach & intestine metastasized by BLCA cells from mice bearing orthotopic xenograft from **Figure 2E**. Metastatic positive organs is based on observed ex-vivo BLI signal. The number of mice showing metastasis is indicated in the numerator whereas the total number of mice in the group is indicated in the denominator in each cell. **B)** IHC analysis of SMARCB1 in *ex vivo* metastatic organs (representative images from lung and intestine) derived from orthotopic SMARCB1 KO (clone 16) xenografts (metastatic organ derived from xenografts as shown in **Figure 2D**). Red arrows indicate negative expression of SMARCB1 in SMARCB1 KO tumor cells. Higher magnification images (denoted in small boxes) show the tumor cells with loss of SMARCB1. Scale bar represents 100μm. C16 indicates clone16.

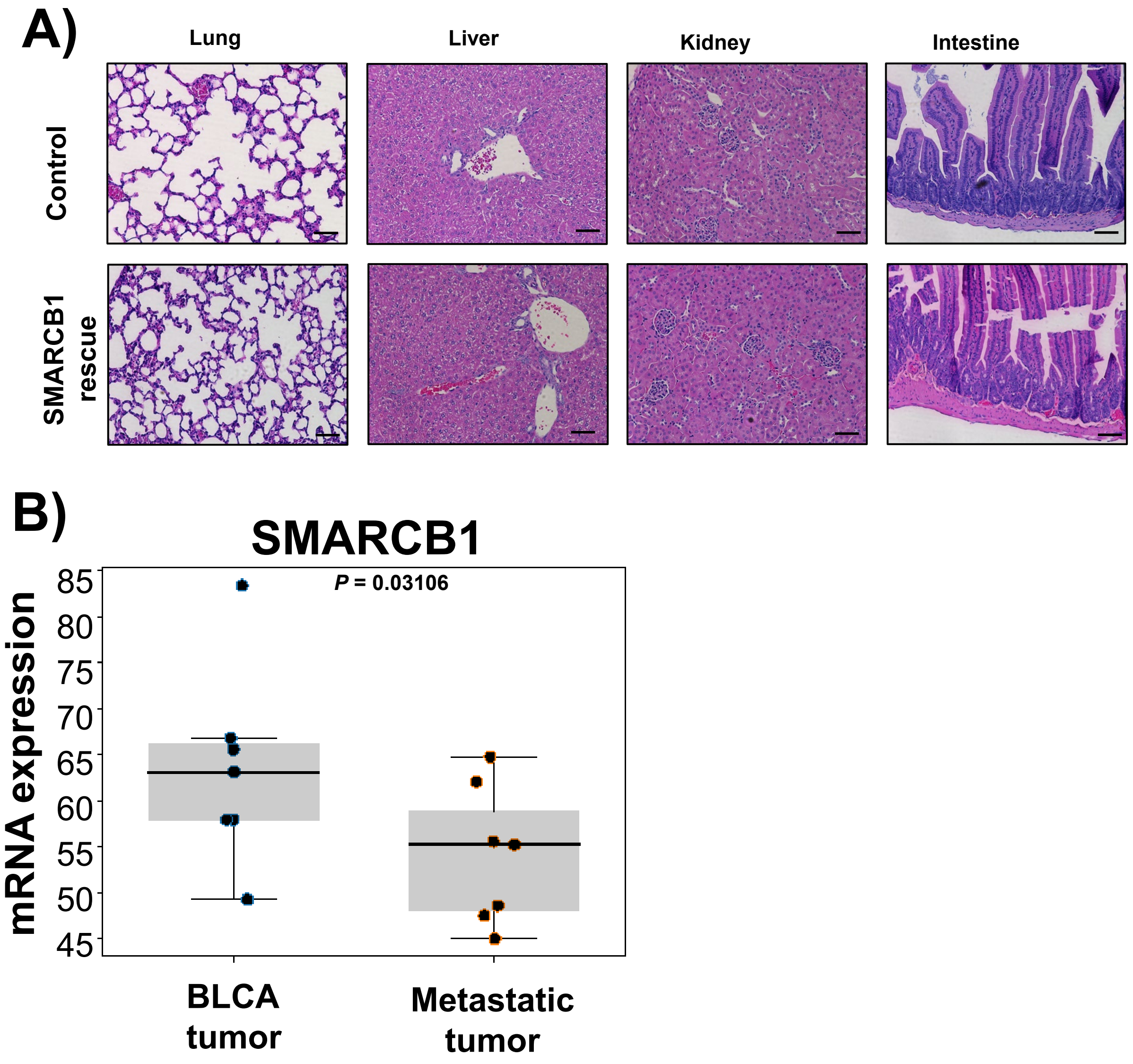

Supplementary Figure 7. H & E analysis of control and SMARCB1 rescue xenograft tumors and SMARCB1 mRNA expression in primary and matched metastatic BLCA patient tumors. A) Histology images of H&E staining of *ex vivo* metastatic organs derived from Ctrl and SMARCB1 rescue orthotopic T24 xenografts (clone 16) derived from experiment as shown in **Figure 2**. Scale bar represents 100μm. **B)** RNA expression of SMARCB1 from RNA-sequencing in FFPE specimens from primary BLCA tumors (n=7) and corresponding matched metastatic tumors (n=7). Data obtained in collaboration with National Cancer Institute (NCI). mRNA expression from RNA seq (Counts per million) analysis (P = 0.03106; the p value calculation was performed by paired student t-test, (one-tailed) using the SciPy package with python 2.7 version]. Source data are provided as a Source Data file.

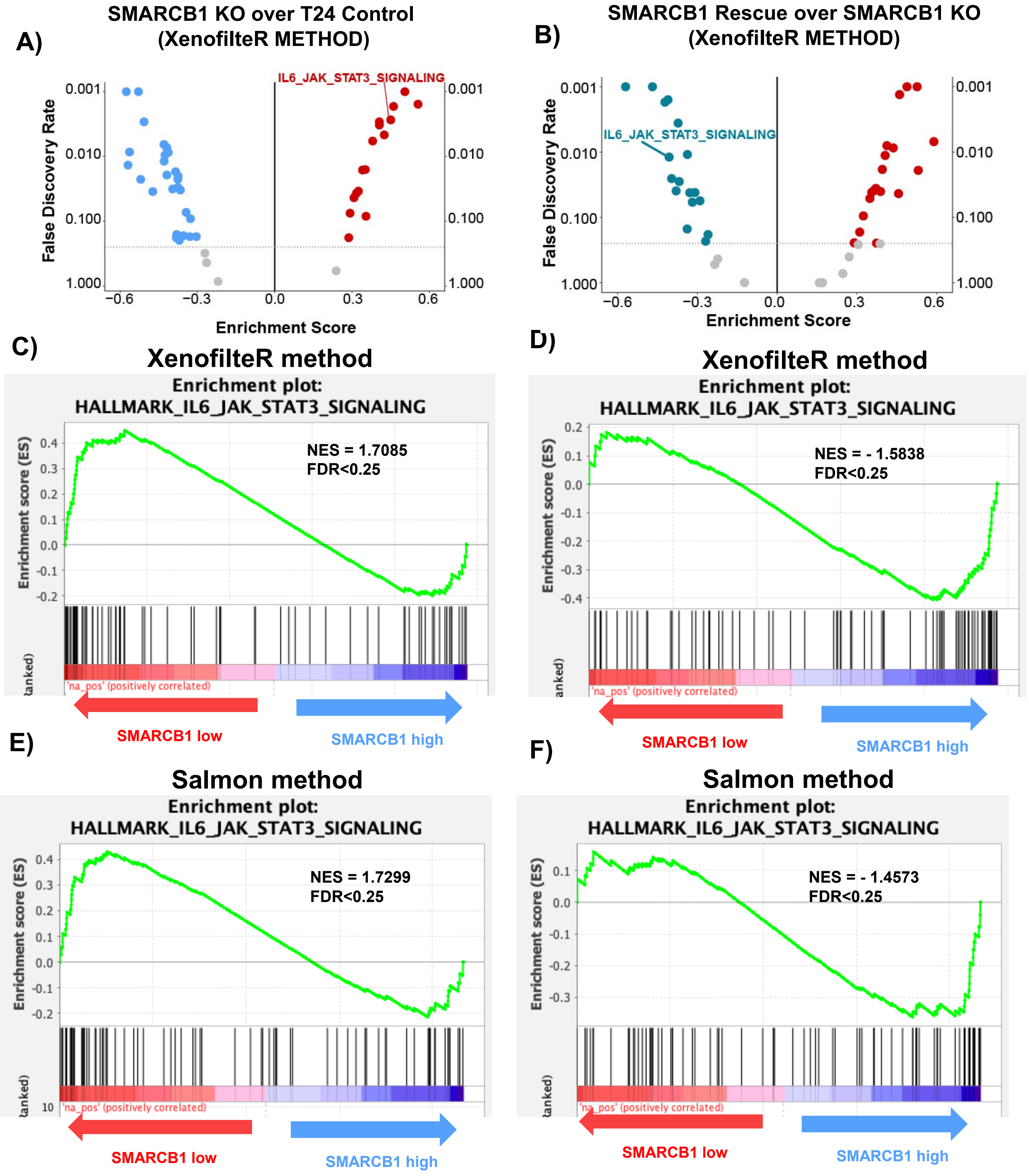

Supplementary Figure 8.

Supplementary Figure 8. IL6/JAK/STAT3 signaling pathway is enriched in SMARCB1 loss tumors using two different (XenofilteR and Salmon) methods. **A-B)** Gene set enrichment analysis (represented by volcano plots) of transcriptomics data from orthotopic tumors (mouse xenograft experiments as shown in Figure 2) derived from T24 control (n=4), SMARCB1 KO (clone 16; n=4), and SMARCB1 rescue (n=4) bladder tumors by XenofilteR method (FDR<0.25). **C-D)** GSEA enrichment plots by XenofilteR method showing IL6/JAK/STAT3 signaling pathways in **C)** SMARCB1 KO over T24 Control (panel C; GSEA plot; NES=1.7085; p<0.0001) and **D)** SMARCB1 rescue over SMARCB1 KO (panel D; GSEA plot; NES=-1.5838; p=0.003). **E-F)** Same as **C-D**, but salmon method shows the activation of IL6/JAK/STAT3 signaling pathway in SMARCB1 KO over T24 Control (panel E; GSEA plot; NES=1.7299; p < 0.0001) and SMARCB1 rescue over SMARCB1 KO (panel F; GSEA plot; NES=-1.4573; p=0.008**)**.

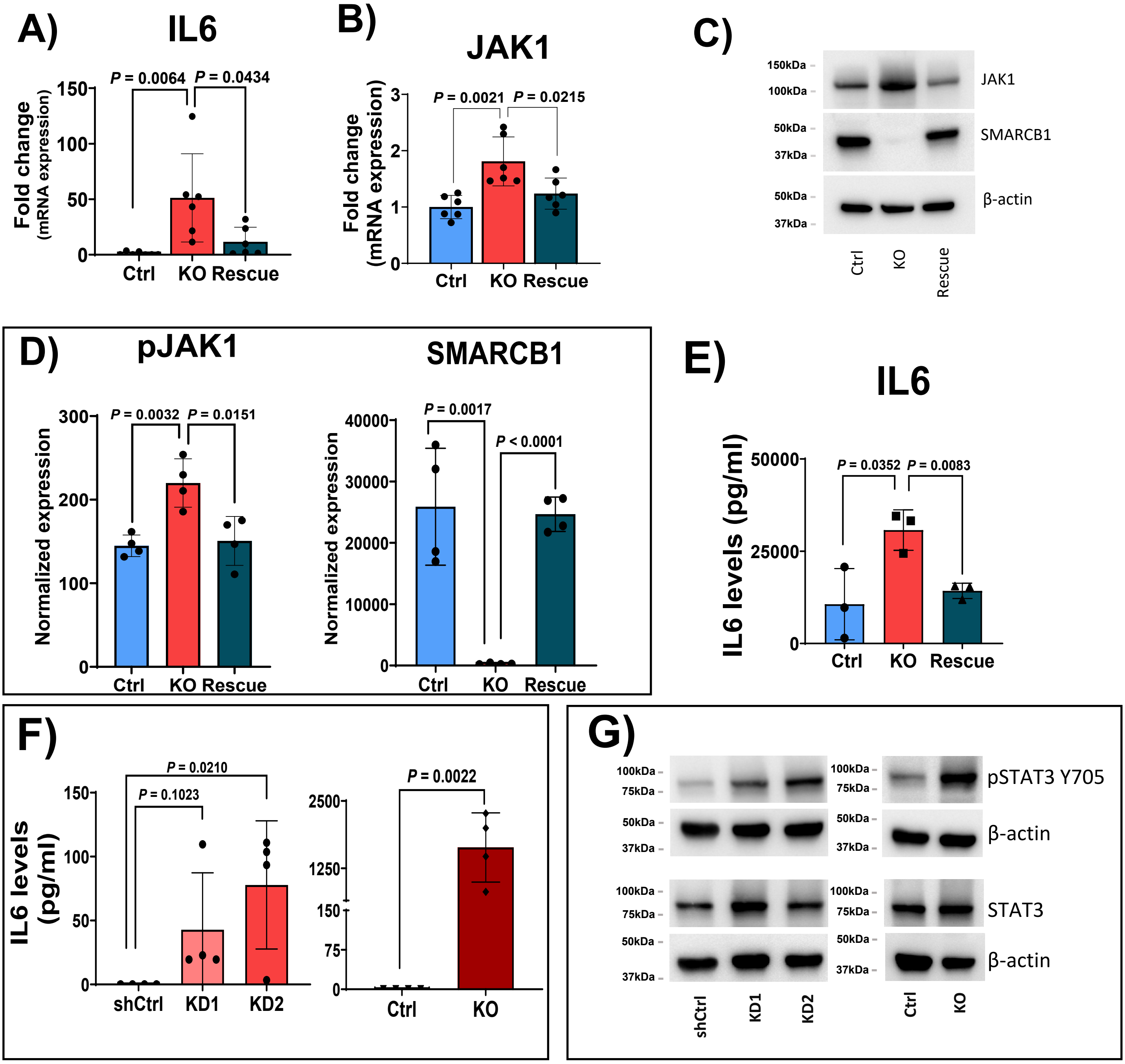

Supplementary Figure 9.

Supplementary Figure 9. SMARCB1 loss enhances the IL6/JAK/STAT3 signaling. A) Relative fold change in mRNA levels of IL6 from T24 ctrl (n=7 replicates, each one analyzed under three technical replicates and sampled across 4 different mice), SMARCB1 KO (n=6 replicates sampled across three different mice; each one analyzed under three technical replicates) and SMARCB1 rescue (n=6 replicates sampled across three different mice; each one analyzed under three technical replicates) orthotopic xenografts. Normalized with housekeeping gene (18S rRNA) [Data are represented as mean ± standard deviation (SD)]. **B)** Relative fold change in mRNA levels of JAK1 from T24 ctrl (n=6 replicates sampled across three different mice; each one analyzed under three technical replicates), SMARCB1 KO (n=6 replicates sampled across three different mice; each one analyzed under three technical replicates) and SMARCB1 rescue (n=6 replicates sampled across three different mice; each one analyzed under three technical replicates) orthotopic xenografts. Normalized with β-actin. [Data are represented as mean ± standard deviation (SD)]. **C)** Immunoblot analysis of total JAK1, SMARCB1 in spheroids derived from T24 ctrl, KO and rescue cell lines. β-actin was used as loading control. **D)** Scatter plot represents RPPA normalized signal intensities for pJAK1(Y1034/35) and SMARCB1 in protein lysates derived from T24 ctrl, KO, and rescue spheroids (n=4 biological replicates) [Data are represented as mean ± standard deviation (SD)]. **E)** Quantitation of IL6 secretion by Enzyme-Linked Immunoassay (ELISA) in T24 ctrl, SMARCB1 KO, and SMARCB1 rescue BLCA cell lines (n=3 biological replicates) [Data are represented as mean ± standard deviation (SD)]. **F)** Quantitation of IL6 secretion by enzyme-linked immunoassay (ELISA) in T24 shCtrl, SMARCB1 KD1, SMARCB1 KD2, T24 control and SMARCB1 KO derived plasma from mice (n= 4 biological replicates) [Data are represented as mean ± standard deviation (SD)]. **G)** Immunoblot analysis showing the expression levels of pSTAT3 (Y705) and STAT3 in SMARCB1 KD and SMARCB1 KO derived tumors. Same lysate was used for top and bottom panels for panel **G**. For panels **A, B, D, E,** and **F** *P* values were determined by unpaired two-tailed Student's *t* test. Source data are provided as a Source Data file.

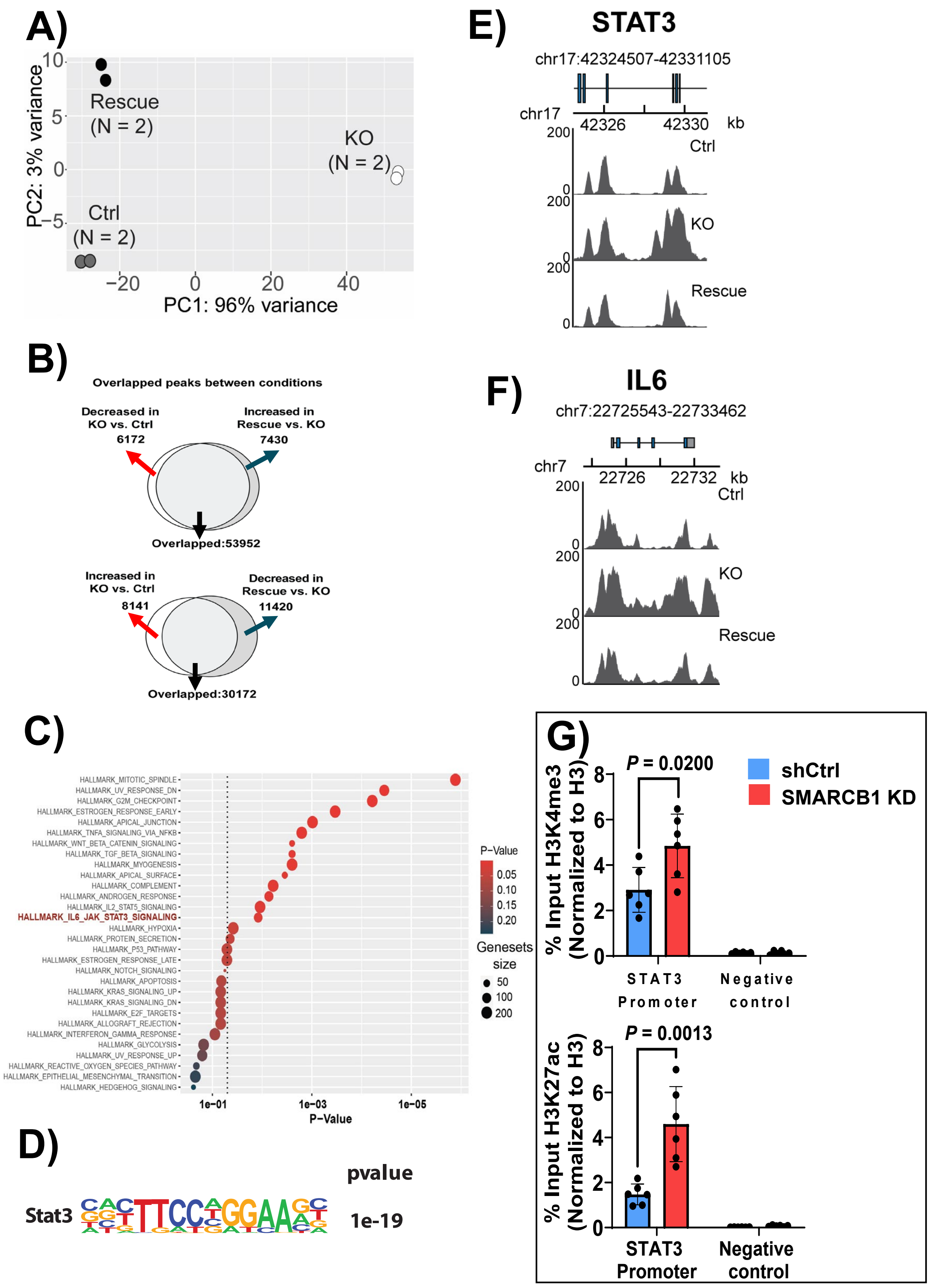

Supplementary Figure 10.

Supplementary Figure 10. Genome-wide changes in the SMARCB1 KO and rescue BLCA cell lines A) PCA plot demonstrating genome-wide changes of accessibility in WT (n=2 biological replicates), KO (n=2 biological replicates) and rescue (n=2 biological replicates). **B)** Venn diagram showing the overlapped peaks identified by ATAC-seq between SMARCB1 KO over ctrl and SMARCB1 rescue over SMARCB1 KO (Top Venn diagram shows the decreased peaks in KO over ctrl and increased in rescue over KO; Bottom Venn diagram shows the increased peaks in KO over ctrl and decreased in rescue over KO). **C)** Ranking of hallmark gene sets that have increased accessibility in ATAC-seq upon loss of SMARCB1. **D)** Enrichment of STAT3 motifs from sites with increased accessibility in KO. **E)** Genome track demonstrating increased accessibility of the STAT3 locus upon SMARCB1 KO that is reversed upon SMARCB1 rescue. **F)** Genome track demonstrating increased accessibility of the IL6 locus upon SMARCB1 KO, reversed upon SMARCB1 rescue. **G)** ChIP-qPCR analysis shows increased levels of H3K27ac and H3K4me3 on STAT3 promoter in SMARCB1-knockdown (KD2) xenografts (three biological replicates; each one analyzed under two technical replicates; n=6) [Data are represented as mean ± standard deviation (SD)]. For panel **G**, *P* values were determined by unpaired two-tailed Student's *t* test. Source data are provided as a Source Data file.

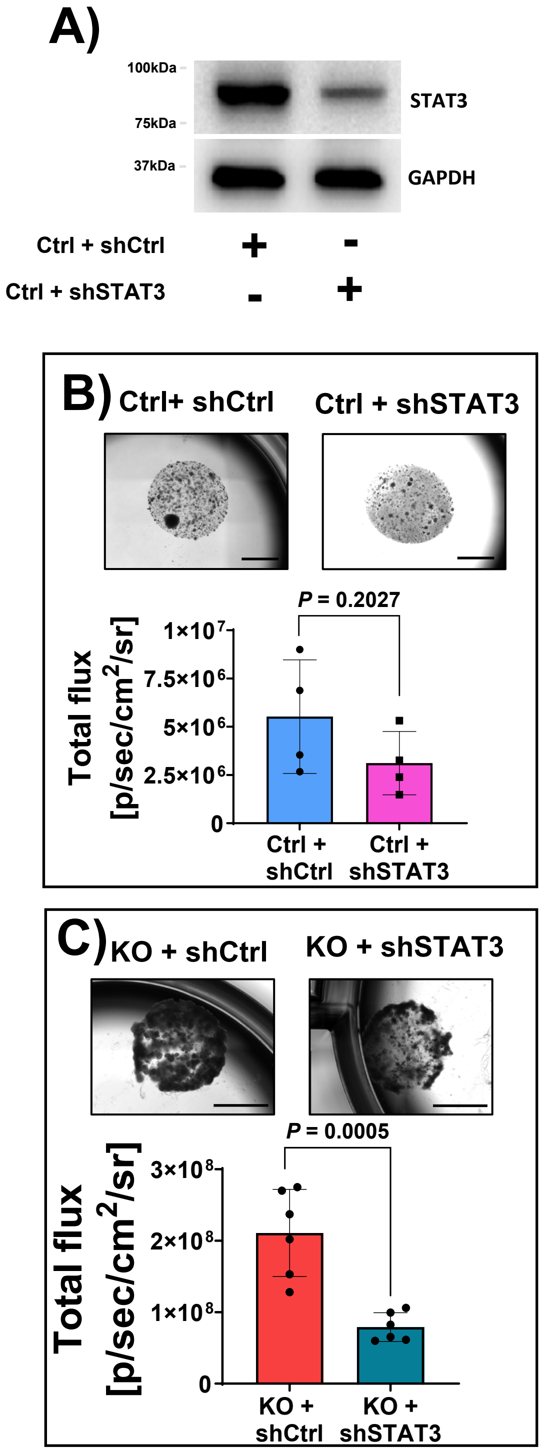

Supplementary Figure 11. Spheroid growth in T24 controls and SMARCB1 KO upon shSTAT3. A) Immunoblot showing the confirmation of shSTAT3 in T24 control BLCA cell lines. GAPDH was used as loading control. **B)** Spheroid assay of luciferase labelled T24 ctrl cell line with shSTAT3 at day 15 (n=4 replicates). Scale bar represents 2000μm. On day 15, measurement of luciferase by BLI signal showed no difference in total flux between T24 Ctrl with shSTAT3 compared to T24 Ctrl with shCtrl spheroids [Data are represented as mean ± standard deviation (SD)]. Images were captured at 9 different focal points and stitched together by Biotek Gen5 software. **C)** Spheroid assay in luciferase labelled SMARCB1 KO with shCtrl and shSTAT3 on day 8 (n=6 replicates). Scale bar represents 2000μm. On day 8, measurement of luciferase by BLI imaging signal showed decreased total flux between T24 SMARCB1 KO with shSTAT3 compared to T24 SMARCB1 KO with shCtrl spheroids [Data are represented as mean ± standard deviation (SD)]. Images were captured at 4 different focal points and stitched together by Biotek Gen5 software. For panels **B** and **C**, *P* values were determined by unpaired two-tailed Student's *t* test. Source data are provided as a Source Data file.

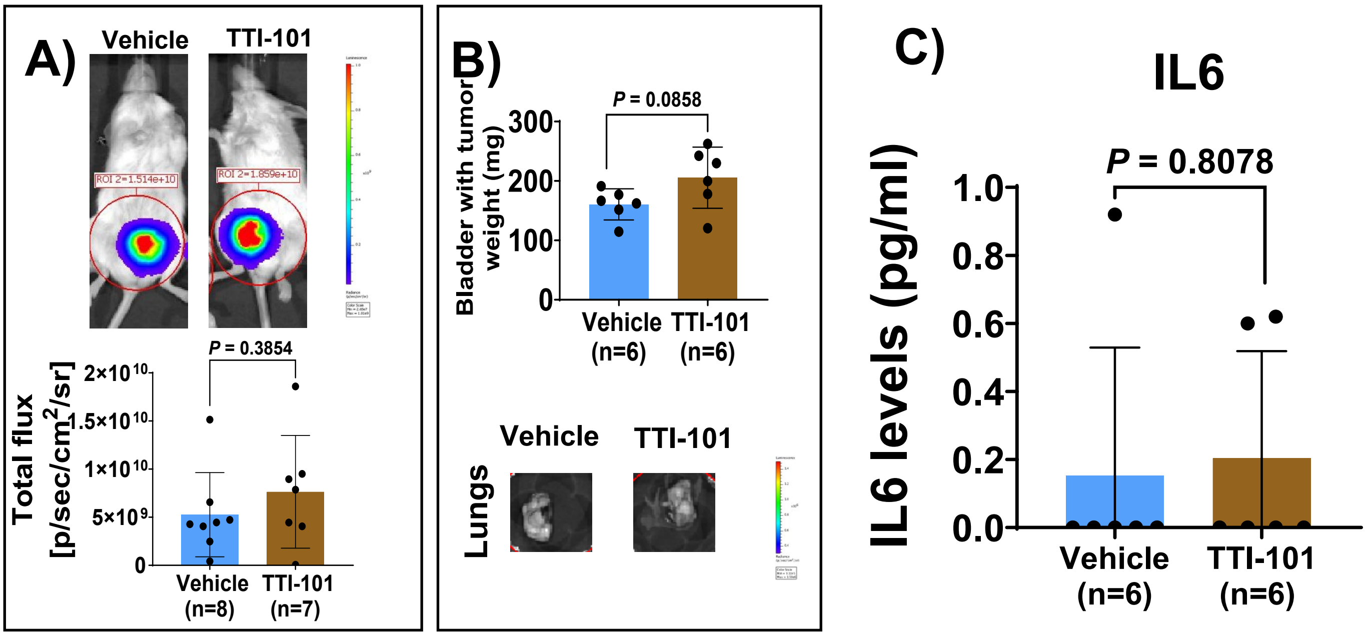

Supplementary Figure 12. Effect of TTI-101 in T24 control orthotopic BLCA xenografts. A) Top panel shows representative mice harboring luciferase labeled T24 control cells were treated with TTI-101. Bottom panel scatter plot represents the quantification of total flux measured by BLI signal (on day 35) on vehicle (n=8) and TTI-101 (n=7) [Data are represented as mean ± standard deviation (SD)]. **B)** Top panel shows scatter plot representing weight of bladder tumor (at end point; day 37) in vehicle (n=6) and TTI-101 (n=6). (Note: 2 mice from vehicle and 1 mouse from TTI-101 group were died before end point). [Data are represented as mean ± standard deviation (SD)]. Bottom panel shows representative ex vivo BLI images of metastatic lesions (lungs) of vehicle, TTI-101 BLCA xenografts. BLI signal was not observed for both vehicle and TTI-101 indicating no metastatic growth. **C)** Quantification of IL6 secretion by ELISA in T24 control xenografts treated with vehicle (n=6) and TTI-101 (n=6) inhibitor [Data are represented as mean ± standard deviation (SD)]. For panels **A, B** and **C**, *P* values were determined by unpaired two-tailed Student's *t* test. Source data are provided as a Source Data file.

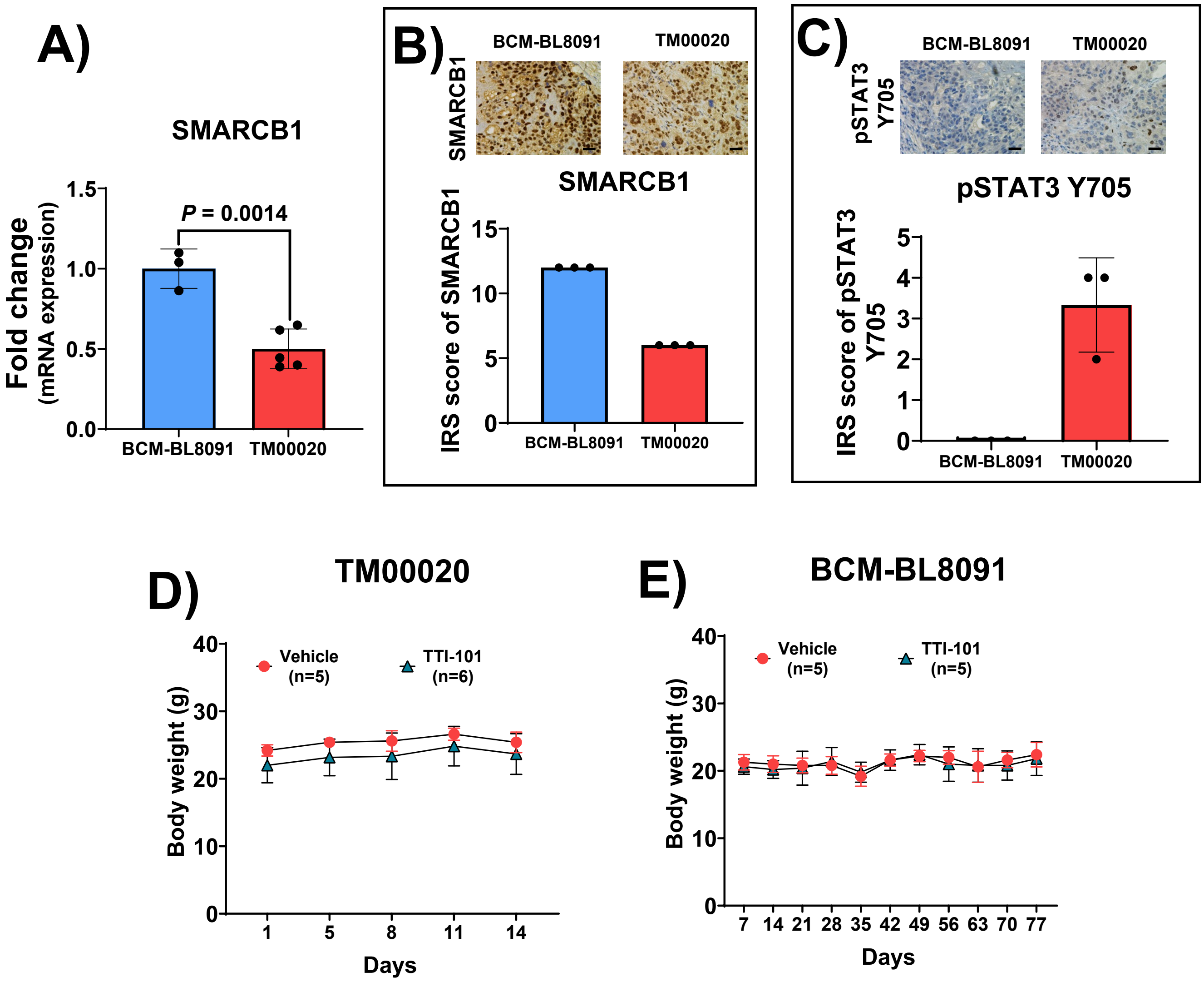

Supplementary Figure 13. Characterization of BLCA PDXs used in this study. A) Fold change of SMARCB1 mRNA expression from BCM-BL8091 (n=3 biological replicates each one analyzed under three technical replicates) and TM00020 (n=5 biological replicates each one analyzed under three technical replicates) PDX tumors. Normalized with GAPDH [Data are represented as mean ± standard deviation (SD)]. **B)** BLCA patient-derived xenografts (PDX) BCM-BL8091 PDX showing high expression of SMARCB1 [Immunoreactive score (IRS score=12; BCM-BL8091; n=3 replicates] and TM00020 showing low SMARCB1 expression (IRS score=6; TM00020; n=3 replicates). The nuclear expression of SMARCB1 was used for IHC analysis [Data are represented as mean ± standard deviation (SD)]. **C)** BLCA patient-derived xenografts (PDX) BCM-BL8091 showing low pSTAT3 (Y705) (IRS score=0; BCM-BL8091; n=3 replicates) and TM00020 showing high pSTAT3 (Y705) expression (IRS score=2-4; TM00020; n=3 replicates). The nuclear expression of pSTAT3 (Y705) was used for IHC analysis [Data are represented as mean ± standard deviation (SD)]. **D)** Line graphs represent the body weights of SMARCB1-deficient PDX (TM00020) treated with vehicle (n=5) and TTI-101 (n=6) across 14 days [Data are represented as mean ± standard deviation (SD)]. **E)** Line graphs represent the body weights of SMARCB1 high PDX (BCM-BL8091) treated with vehicle (n=5) and TTI-101 (n=5) across 77 days [Data are represented as mean ± standard deviation (SD)]. For panel **A**, *P* values were determined by unpaired two-tailed Student's *t* test. For panels **B** and **C**, scale bar represents 100 µm. Source data are provided as a Source Data file.

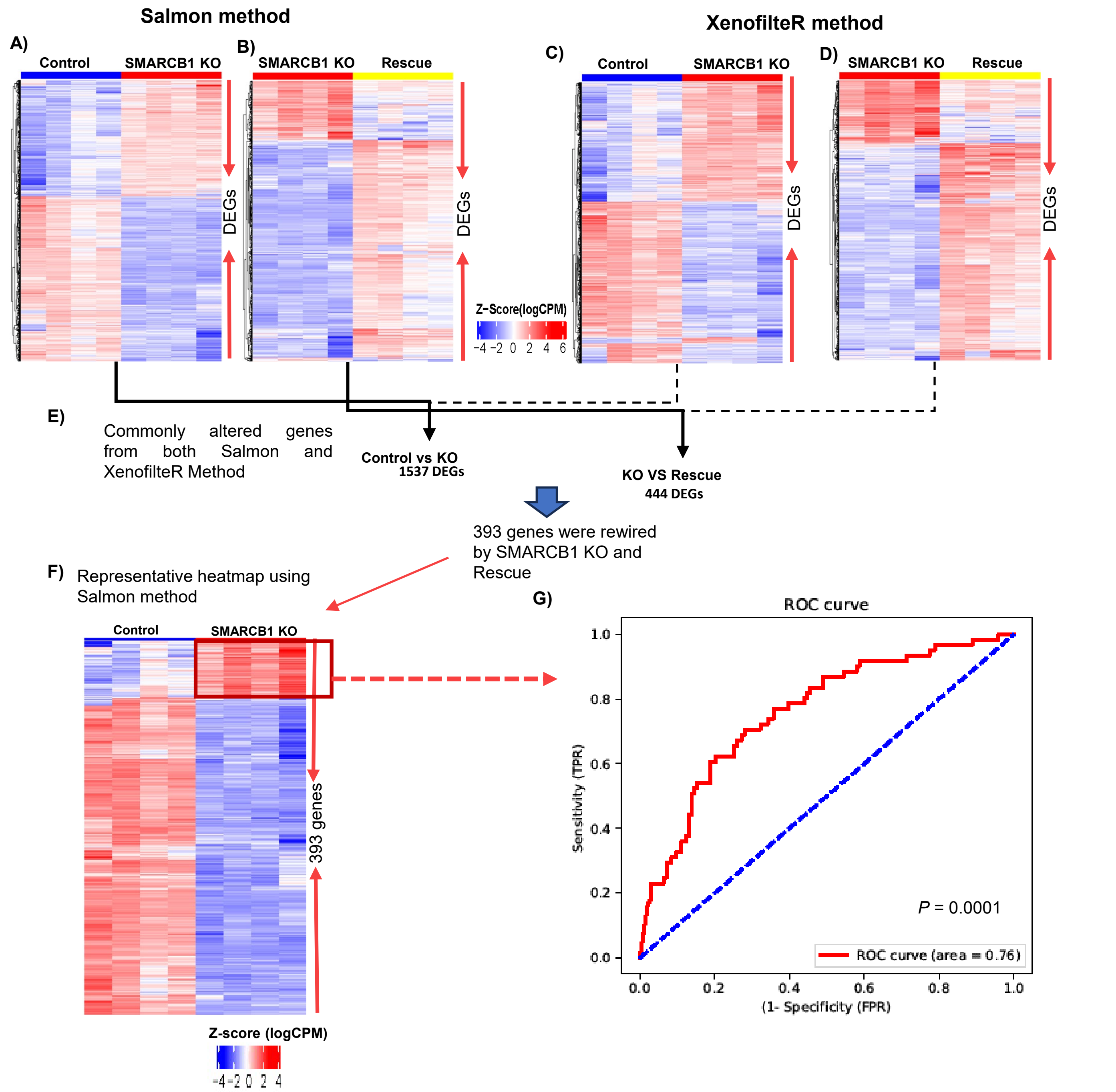

Supplementary Figure 14. Identification of transcriptional signature to predict SMARCB1-deficient BLCA tumors. A-D) Transcriptomic analysis was performed using Salmon (panel A-B) and XenofilteR (panel C-D) methods. **A)** Heat map represents the comparison between T24 control vs SMARCB1 KO (n=4; FDR<0.05 and FC >2) using Salmon method. **B)** Heat map represents the comparison between SMARCB1 KO vs SMARCB1 rescue (n=4; FDR<0.05 and FC >2) using Salmon method. **C)** Same as A, but XenofilteR method between T24 control vs SMARCB1 KO (n=4; FDR<0.05 and FC >2). **D)** Same as B but XenofilteR method between SMARCB1 KO vs SMARCB1 rescue (n=4; FDR<0.05 and FC >2). **E)** Identification of common (Salmon and XenofilterR) differentially expressed genes by SMARCB1 loss (between panel A and C) and SMARCB1 rescue (between panel B and D). A total of 1537 genes are common differentially expressed between T24 control vs SMARCB1 KO; 444 genes are common differentially expressed between SMARCB1 KO vs rescue using both Salmon and XenofilteR methods. Among them 393 genes were rewired by SMARCB1 loss and gain using both Salmon and XenofilteR methods (**Supplementary Data 10**). **F)** A representative heatmap of 393 genes (59 genes are upregulated and 334 genes are downregulated: a total of 393) by SMARCB1 loss (control vs KO) using Salmon method (Note: Heatmap was generated from Panel A). Red box indicates 59 genes increased by SMARCB1 KO and decreased by SMARCB1 rescue (**Supplementary Data 10**). **G)** Receiver operator characteristic (ROC) curve analysis showing the prediction of SMARCB1-deficient with activation of pSTAT3 (n=110; Refer to **Fig. 1E**) vs. other patients (n=224; Refer to **Fig.1E**) in BLCA patients. A set of 55 out of 59-gene signature were used for analysis (derived from SMARCB1 KO xenografts) and yielded area under the ROC (AUC) of 0.76; *P*=0.0001.

Supplementary Table 1. sgRNA sequences used for generating SMARCB1 knockout**.**

Gene	Primer sequence (5'-3')
SMARCB1 -sgRNA1	TGAGAACGCATCTCAGCCCG
SMARCB1- sgRNA2	CATCGATCTCCATGTCCAGC
Non-targeting control	GGGACGCGAAAGAAACCAGT

Supplementary Table 2. PCR primers are used for confirmation of genomic DNA deletion for this study.

Gene	Primer sequence (5'-3')
SMARCB1-FWD.Primer	CGATTCTATTGCAGACAG
SMARCB1-REV.Primer	GACTGAACCCAGAGCCCAC
Sequencing primer	CCACGTAACACACAGGGGT

Note: FWD.= Forward; REV. = Reverse.

Supplementary Table 3. List of antibodies used for this study.

Antibody name	Catalogue number	Vendor	Dilution for western blot	Dilution for IHC	ChIP
SMARCB1/BAF47	91735	Cell signaling	1:3000		*
JAK1	50996S	Cell signaling	1:1000		
STAT3	12640	Cell signaling	1:1000		
SMARCA4/BRG1	52251	Cell signaling	1:1000		
SMARCC2/BAF170	12760	Cell signaling	1:1000		
SMARCC1/BAF155	11956	Cell signaling	1:1000		
pSTAT3 (Tyr705)	9145	Cell signaling	1:500		
Beta-actin	3700	Cell signaling	1:5000		
GAPDH	5174	Cell signaling	1:3000		
Anti-Mouse IgG	7076	Cell signaling	1:3000		
Anti-Rabbit IgG	7074	Cell signaling	1:3000		
pSTAT3 (Tyr705)	SC-8059	Santha Cruz		1:50	
SMARCB1	612110	BD Biosciences		1:200	
H3K4Me3	AB8580	abcam			*
H3K27Ac	AB4729	abcam			*
H3	AB1791	abcam			*

*** indicates the antibody used for ChIP-qPCR. The ChIP-qPCR was performed at Epigenomics profiling core at MD Anderson and the antibody dilutions were used as per their standard operational procedures.**

Supplementary Table 4. Real-time PCR Primers were used for this study.

Gene	Primer sequence (5'-3')
SMARCB1-FWD. Primer	GGCATCAGAAGACCTACGCCTT
SMARCB1-REV. Primer	CTCCATCTCAGCGTCTGTCAGA
STAT3 -FWD. Primer	CTTTGAGACCGAGGTGTATCACC
STAT3-REV. Primer	GGTCAGCATGTTGTACCACAGG
JAK1 FWD. Primer	TGGAGGTAACCACATAGC
JAK1 REV. Primer	CCGAGAACCCAAATAGTC
beta-actin-FWD. Primer	AGCGGGAAATCGTGCGTG
beta-actin-REV. Primer	GGGTACATGGTGGTGCCG
GAPDH-FWD. Primer	TCAAGGCTGAGAACGGGAAG
GAPDH-REV. Primer	CGCCCCACTTGATTTTGGAG
**Gene**	**Catalog Number**
IL6	Hs00985639_m1 (Invitrogen; Taqman Probe)
18S rRNA	4310893E (Thermofisher scientific; Taqman Probe)

Note: FWD. = Forward; REV. = Reverse.

**Supplementary Table 5.** CHIP-qPCR Primer sequences were used for this study.	
**ChIP**	**Primers**
H3K27Ac/H3K4Me3/SMARCB1	SNF5-STAT3 Promoter-FWD.Primer: CGGAATGTCCTGCTGAAAAC
	SNF5-STAT3 Promoter-REV.Primer: CAGGAGGGAGCTGTATCAGG
	H3K27ac Neg-STAT3 Promoter-FWD.Primer: ATGCAGTGGCATGATCTCAG
	H3K27ac Neg-STAT3 Promoter-REV.Primer: CAGGTGGTTAGGTGGAAGGA
	SNF5 Neg-STAT3 Promoter-FWD.Primer: TGGTTGAAATGGATGGAAAG
	SNF5 Neg-STAT3 Promoter-REV.Primer: GGCACATAGATGCCTGAACA
Note: FWD.= Forward ; REV.= Reverse.; Neg- Negative Control region	

**Supplementary Table 6.** shRNA sequences for generating shCtrl, SMARCB1 and STAT3 knockdown for this study.	
**Gene**	**Sequence/Catalogue number**
Control shRNA	ATCTCGCTTGGGCGAGAGTAAG
STAT3 shRNA	TGACTTTGATTTCAACTAT
**Gene**	**Catalogue number**
Control shRNA	SHC002 (SIGMA)
SMARCB1 shRNA KD1	TRCN0000308015 (SIGMA)
SMARCB1 shRNA KD2	TRCN0000295966 (SIGMA)
