# Supplementary material for: The IL6/JAK/STAT3 signaling axis is a therapeutic vulnerability in SMARCB1-deficient bladder cancer
Source: Nat Commun. 2024 Feb 14;15:1373. doi: 10.1038/s41467-024-45132-2 (PMC10867091; doi:10.1038/s41467-024-45132-2)
Supplement: Supplementary file 3 — Description of Additional Supplementary Data [file 41467_2024_45132_MOESM3_ESM.docx]

**Description of Additional Supplementary Files**

**Supplementary Data 1.**

Description: **A).** Patient identifiers, SMARCB1 high and low expression, which were used for Kaplan-Meier plot from TCGA-BLCA to generate **Fig. 1A**. **B).** Table represents the TCGA-BLCA data used to generate **Fig. 1E**.

**Supplementary Data 2.**

Description: Gene set enrichment analysis (GSEA) associated with SMARCB1 low and SMARCB1 high from the TCGA-BLCA cohort. HALLMARK_IL6_JAK_STAT3_SIGNALING was highlighted in orange and shown in **Fig.1B.**

**Supplementary Data 3.**

Description: Gene set enrichment analysis (GSEA) associated with SMARCB1 low and SMARCB1 high in the GSE48276 (Choi), GSE32548 (Lindgren) and GSE31684 (Riester) BLCA cohorts. HALLMARK_IL6_JAK_STAT3_SIGNALING was highlighted in orange and represented in **Supplementary Fig. 2A-C.**

**Supplementary Data 4.**

Description: Gene set enrichment analysis (GSEA) associated with SMARCB1 shallow/deep deletion versus SMARCB1 diploid in TCGA cohort and HALLMARK_IL6_JAK_STAT3_SIGNALING was represented in **Supplementary Fig. 2D**.

**Supplementary Data 5.**

Description: **A).** Pan cancer analysis (Excluding BLCA) of SMARCB1 copy number alterations derived from TCGA cohort. mRNA expression levels of corresponding patients were shown as rpkm or log2rpkm. A total of 31 cancers were shown. **B).** Table representing the percentage of SMARCB1 copy number alterations in each cancer type from TCGA data set.

**Supplementary Data 6.**

Description: Gene set enrichment analysis (GSEA) associated with SMARCB1 in T24 derived orthotopic xenografts and mapping percentage of human and mouse. Analysis was performed using Salmon and XenofilteR methods which are enriched in key hallmark pathways. **A).** GSEA associated with SMARCB1 knockout and SMARCB1 control which are enriched in both methods (Salmon and XenofilteR). **B).** GSEA associated with SMARCB1 rescue and SMARCB1 knockout which are enriched in both methods (Salmon and XenofilteR). HALLMARK_IL6_JAK_STAT3_SIGNALING was represented in **Supplementary Fig.8C-F**. **C).** Mapping percentage of human and mouse reads from RNA-seq data derived BLCA xenografts.

**Supplementary Data 7.**

Description: List of hallmark gene sets that have increased accessibility in ATAC-seq upon loss of SMARCB1 (Refer to **Supplementary Fig.10C**). IL6-JAK-STAT3 signaling pathway was highlighted. The enrichment of gene sets from increased sites was performed using hypergeometric tests.

**Supplementary Data 8.**

Description: ATAC-seq analysis represents the list of increased motifs compared to unchanged sites upon SMARCB1 KO in T24 BLCA cell line (Note: STAT3 motif was represented in **Supplementary Fig.10D**). P values for motif over-representation were calculated using HOMER with default parameters.

**Supplementary Data 9.**

Description: List of bladder cancer PDX models available in Jackson lab with SMARCB1 copy number alterations and corresponding mRNA expression levels. TM00020 (highlighted) PDX model was used for the current study.

**Supplementary Data 10.**

Description: List of rewired genes identified based on the integration of SMARCB1 KO over control and rescue over KO using Salmon and XenofilteR methods used to generate **Supplementary Fig. 14F**. List of 55 genes that were used for ROC (**Supplementary Fig. 14G**) were highlighted in Red.
