# Supplementary material for: The IL6/JAK/STAT3 signaling axis is a therapeutic vulnerability in SMARCB1-deficient bladder cancer
Source: Nat Commun. 2024 Feb 14;15:1373. doi: 10.1038/s41467-024-45132-2 (PMC10867091; doi:10.1038/s41467-024-45132-2)
Supplement: Supplementary file 14 — Reporting Summary [file 41467_2024_45132_MOESM14_ESM.pdf]

nature portfolio

Corresponding author(s): Pavlos Msaouel
Shyam M Kavuri
Nagireddy Putluri

Last updated by author(s): 09-27-2023

Reporting Summary

Nature Portfolio wishes to improve the reproducibility of the work that we publish. This form provides structure for consistency and transparency in reporting. For further information on Nature Portfolio policies, see our Editorial Policies and the Editorial Policy Checklist.

Statistics

For all statistical analyses, confirm that the following items are present in the figure legend, table legend, main text, or Methods section.

n/a	Confirmed	
☐	☒	The exact sample size (*n*) for each experimental group/condition, given as a discrete number and unit of measurement
☐	☒	A statement on whether measurements were taken from distinct samples or whether the same sample was measured repeatedly
☐	☒	The statistical test(s) used AND whether they are one- or two-sided<br>*Only common tests should be described solely by name; describe more complex techniques in the Methods section.*
☒	☐	A description of all covariates tested
☒	☐	A description of any assumptions or corrections, such as tests of normality and adjustment for multiple comparisons
☒	☐	A full description of the statistical parameters including central tendency (e.g. means) or other basic estimates (e.g. regression coefficient) AND variation (e.g. standard deviation) or associated estimates of uncertainty (e.g. confidence intervals)
☒	☐	For null hypothesis testing, the test statistic (e.g. *F*, *t*, *r*) with confidence intervals, effect sizes, degrees of freedom and *P* value noted<br>*Give P values as exact values whenever suitable.*
☒	☐	For Bayesian analysis, information on the choice of priors and Markov chain Monte Carlo settings
☒	☐	For hierarchical and complex designs, identification of the appropriate level for tests and full reporting of outcomes
☒	☐	Estimates of effect sizes (e.g. Cohen's *d*, Pearson's *r*), indicating how they were calculated

Our web collection on statistics for biologists contains articles on many of the points above.

Software and code

Policy information about availability of computer code

Data collection	RNA seq was performed on a NovaSeq6000 (Illumina Inc.); ATAC-seq was performed Paired-end sequencing was performed on an Illumina NextSeq 500. qPCR was performed on Quant Studio TM 3 System(Applied Biosystems). Described in methods section.
Data analysis	RNA seq was analyzed using the R package Tximport (v 1.16.1). ATAC-seq reads were mapped using Bowtie 2.1.0 and analyzed, peak calling was performed by MACS 2.1.1, Transcription factor motif enrichment was measured using HOMER (version 4.11). Described in methods section.

For manuscripts utilizing custom algorithms or software that are central to the research but not yet described in published literature, software must be made available to editors and reviewers. We strongly encourage code deposition in a community repository (e.g. GitHub). See the Nature Portfolio guidelines for submitting code & software for further information.

Data

Policy information about availability of data

All manuscripts must include a data availability statement. This statement should provide the following information, where applicable:

- Accession codes, unique identifiers, or web links for publicly available datasets
- A description of any restrictions on data availability
- For clinical datasets or third party data, please ensure that the statement adheres to our policy

All the raw data was available in supplementary materials. Raw and processed RNASeq data from mouse xenografts are available at GEO Hub with accession

number GSE212762. Private access during review is available using the following token: "yhivkogozxslpen". High-throughput data generated in this study (ATAC-seq) have been deposited in the Gene Expression Omnibus (GEO) database with accession number GSE213964. Private access during review is available using the following token: "ujghqokqptepryj".

Human research participants

Policy information about studies involving human research participants and Sex and Gender in Research.

Reporting on sex and gender	De-identified specimens/data was used in this study. Both Male and Female patients were included.
Population characteristics	We have collected available clinical information for this study.
Recruitment	No recruitment involved in this study.
Ethics oversight	H-35808

Note that full information on the approval of the study protocol must also be provided in the manuscript.

Field-specific reporting

Please select the one below that is the best fit for your research. If you are not sure, read the appropriate sections before making your selection.

☒ Life sciences ☐ Behavioural & social sciences ☐ Ecological, evolutionary & environmental sciences

For a reference copy of the document with all sections, see nature.com/documents/nr-reporting-summary-flat.pdf

Life sciences study design

All studies must disclose on these points even when the disclosure is negative.

Sample size	Sample size was determined using power calucaltions.
Data exclusions	No data was excluded for entire study.
Replication	All the experiments were performed with at least two or more replicates.
Randomization	All in vivo experiments were randomized and performed.
Blinding	Not applicable

Behavioural & social sciences study design

All studies must disclose on these points even when the disclosure is negative.

Study description	N/A
Research sample	N/A
Sampling strategy	N/A
Data collection	N/A
Timing	N/A
Data exclusions	N/A
Non-participation	N/A
Randomization	N/A

Ecological, evolutionary & environmental sciences study design

All studies must disclose on these points even when the disclosure is negative.

Study description	N/A
Research sample	N/A
Sampling strategy	N/A
Data collection	N/A
Timing and spatial scale	N/A
Data exclusions	N/A
Reproducibility	N/A
Randomization	N/A
Blinding	N/A

Did the study involve field work? ☐ Yes ☒ No

Field work, collection and transport

Field conditions	N/A
Location	N/A
Access & import/export	N/A
Disturbance	N/A

Reporting for specific materials, systems and methods

We require information from authors about some types of materials, experimental systems and methods used in many studies. Here, indicate whether each material, system or method listed is relevant to your study. If you are not sure if a list item applies to your research, read the appropriate section before selecting a response.

Materials & experimental systems

n/a	Involved in the study	
☐	☒	Antibodies
☐	☒	Eukaryotic cell lines
☒	☐	Palaeontology and archaeology
☐	☒	Animals and other organisms
☒	☐	Clinical data
☒	☐	Dual use research of concern

Methods

n/a	Involved in the study	
☒	☐	ChIP-seq
☒	☐	Flow cytometry
☒	☐	MRI-based neuroimaging

Antibodies

Antibodies used	SMARCB1, pSTAT3 (Y705), STAT3, beta-actin, SMARCC1, SMARCC2, SMARCA4, GAPDH, Jak1
Validation	These antibodies are validated.

Eukaryotic cell lines

Policy information about cell lines and Sex and Gender in Research

Cell line source(s)	Cell lines used for this study were purchased from ATCC
Authentication	Authentication was confirmed by STR typing.
Mycoplasma contamination	No
Commonly misidentified lines (See ICLAC register)	N/A

Palaeontology and Archaeology

Specimen provenance	N/A
Specimen deposition	N/A
Dating methods	N/A

☐ Tick this box to confirm that the raw and calibrated dates are available in the paper or in Supplementary Information.

Ethics oversight	N/A

Note that full information on the approval of the study protocol must also be provided in the manuscript.

Animals and other research organisms

Policy information about studies involving animals; ARRIVE guidelines recommended for reporting animal research, and Sex and Gender in Research

Laboratory animals	NSG mice (NOD.Cg-Prkdcscid Il2rgtm1Wjl/SzJ; 6-8 weeks old mice were involved); SCID-Beige (6-8 weeks old mice were involved);
Wild animals	None
Reporting on sex	Experiments were performed in male and female mice and reported in the manuscript.
Field-collected samples	N/A
Ethics oversight	AN-7324 (Approved animal protocol)

Note that full information on the approval of the study protocol must also be provided in the manuscript.

ChIP-seq

Data deposition

☐ Confirm that both raw and final processed data have been deposited in a public database such as GEO.

☐ Confirm that you have deposited or provided access to graph files (e.g. BED files) for the called peaks.

Data access links *May remain private before publication.*	N/A
Files in database submission	N/A
Genome browser session (e.g. UCSC)	N/A

Methodology

Replicates	N/A
Sequencing depth	N/A
Antibodies	N/A

Peak calling parameters	N/A
Data quality	N/A
Software	N/A

Flow Cytometry

Plots

Confirm that:

☐ The axis labels state the marker and fluorochrome used (e.g. CD4-FITC).

☐ The axis scales are clearly visible. Include numbers along axes only for bottom left plot of group (a 'group' is an analysis of identical markers).

☐ All plots are contour plots with outliers or pseudocolor plots.

☐ A numerical value for number of cells or percentage (with statistics) is provided.

Methodology

Sample preparation	N/A
Instrument	N/A
Software	N/A
Cell population abundance	N/A
Gating strategy	N/A

☐ Tick this box to confirm that a figure exemplifying the gating strategy is provided in the Supplementary Information.

Magnetic resonance imaging

Experimental design

Design type	N/A
Design specifications	N/A
Behavioral performance measures	N/A

Acquisition

Imaging type(s)	N/A
Field strength	N/A
Sequence & imaging parameters	N/A
Area of acquisition	N/A

Diffusion MRI ☐ Used ☐ Not used

Preprocessing

Preprocessing software	N/A
Normalization	N/A
Normalization template	N/A
Noise and artifact removal	N/A
Volume censoring	N/A

Statistical modeling & inference

Model type and settings: N/A

Effect(s) tested: N/A

Specify type of analysis: ☐ Whole brain ☐ ROI-based ☐ Both

Statistic type for inference (See Eklund et al. 2016): N/A

Correction: N/A

Models & analysis

n/a	Involved in the study
☒	☐ Functional and/or effective connectivity
☒	☐ Graph analysis
☒	☐ Multivariate modeling or predictive analysis

Functional and/or effective connectivity: N/A
