# Supplementary material for: The IL6/JAK/STAT3 signaling axis is a therapeutic vulnerability in SMARCB1-deficient bladder cancer
Source: Nat Commun. 2024 Feb 14;15:1373. doi: 10.1038/s41467-024-45132-2 (PMC10867091; doi:10.1038/s41467-024-45132-2)

Source data associated with Figure 2A, 3D, 3G, 4A & Supplementary Figure 4B, 4D, 4F, 9C, 9G, 11A, 13B, 13C

Raw image for Figure 2A

Western for SMARCB1

Raw image

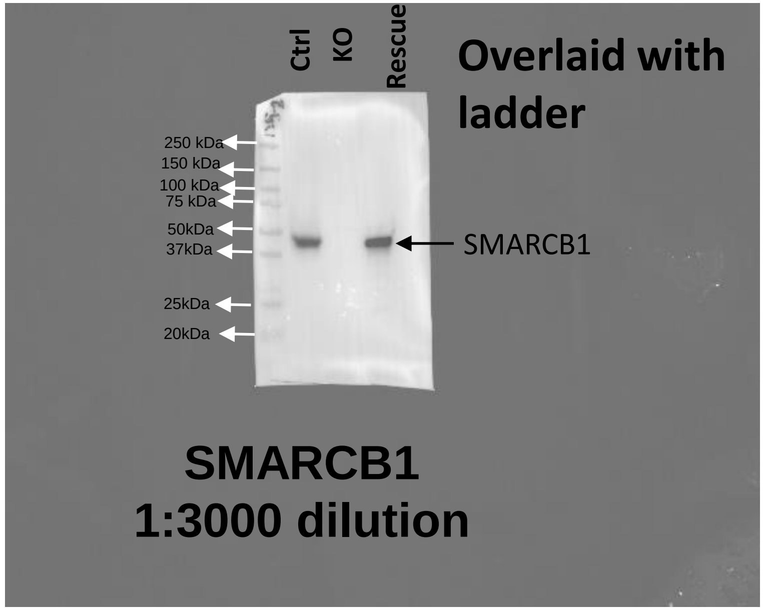

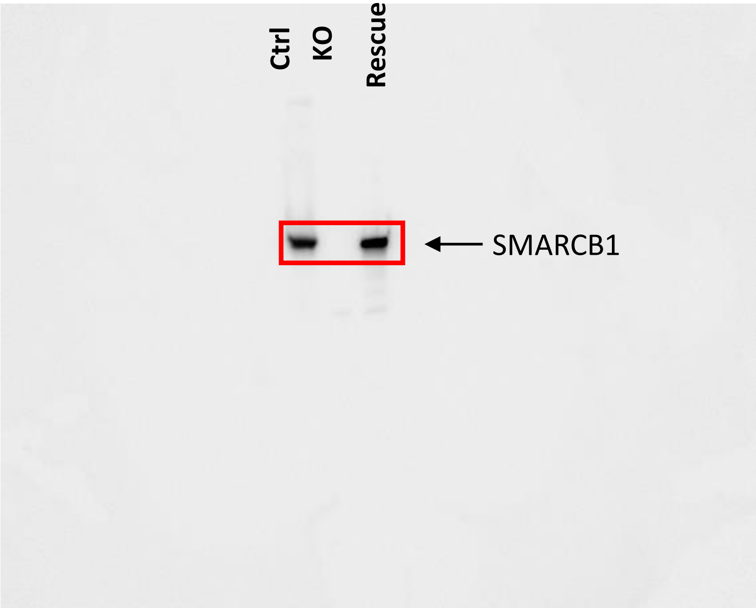

Western for GAPDH

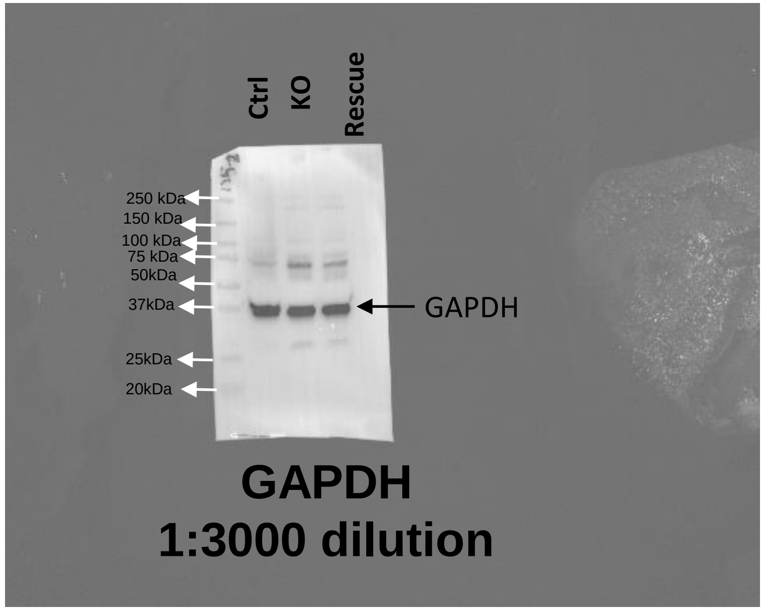

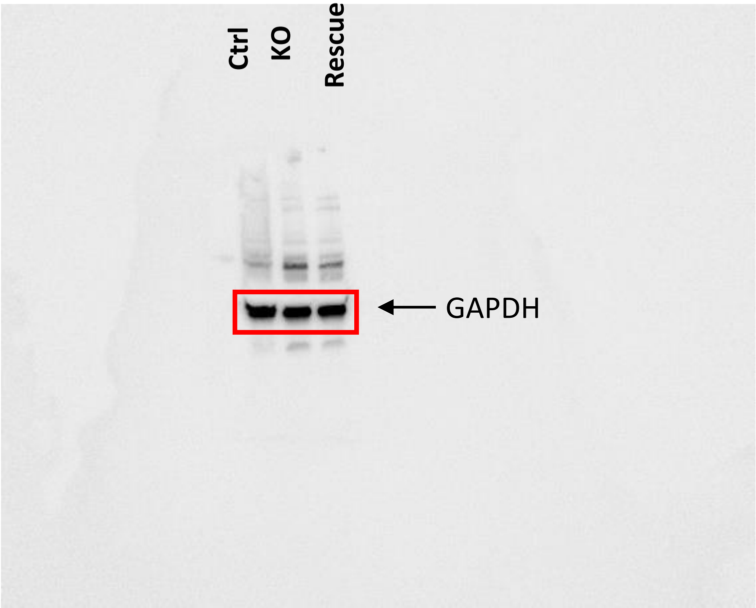

Area used for Figure 2A

SMARCB1 1:3000
GAPDH 1:3000

Blot 125-2

Raw image for Figure 3D- Top panel

Raw image

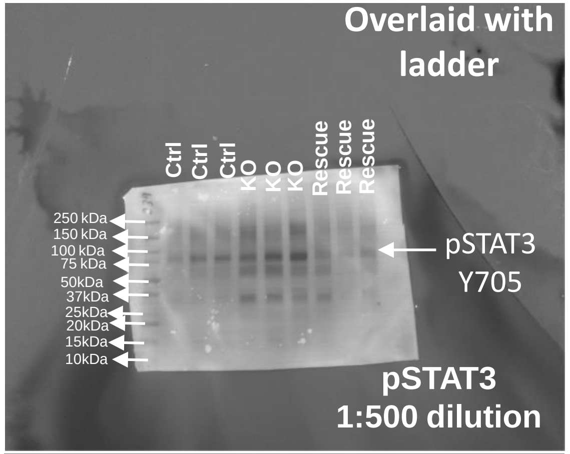

Western for pSTAT3 (Y705)

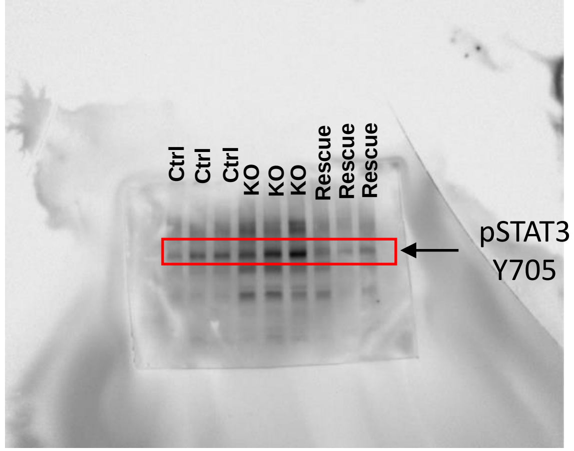

Same protein lysate was used for Blot239 [pSTAT3-Y705, SMARCB1 and beta-actin] and Blot252 [STAT3, SMARCB1 and beta-actin].

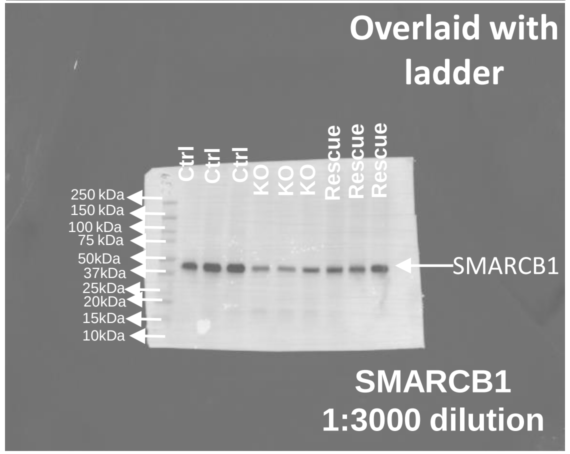

Western for SMARCB1

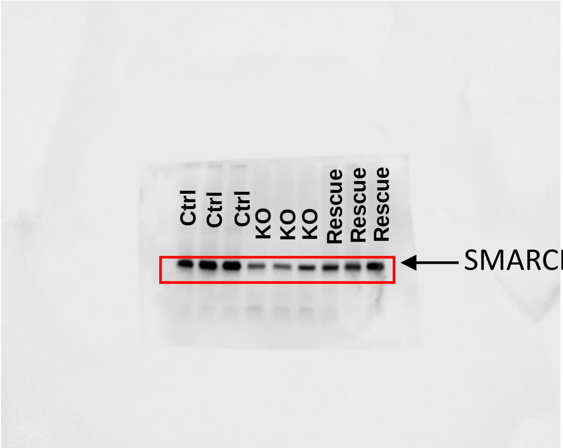

Area used for Figure 3D- Top panel

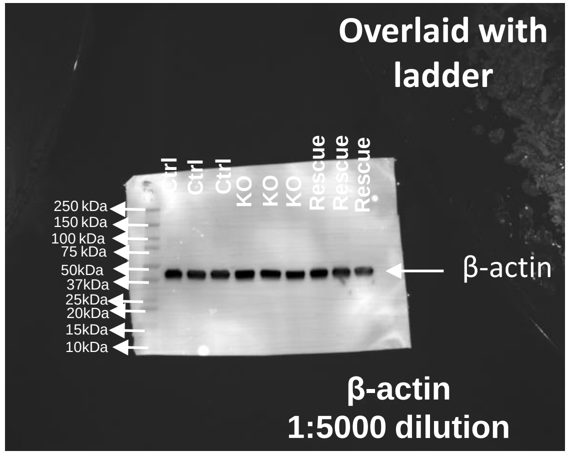

Western for β-actin

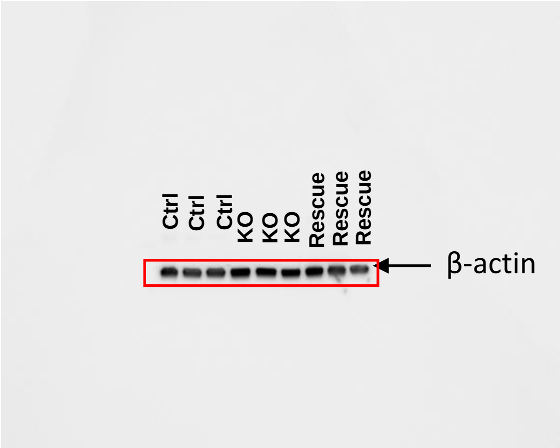

pSTAT3 (Y705) 1:500
SMARCB1 1:3000
β-actin 1:5000

Blot 239

Raw image for Figure 3D-Bottom panel

Raw image

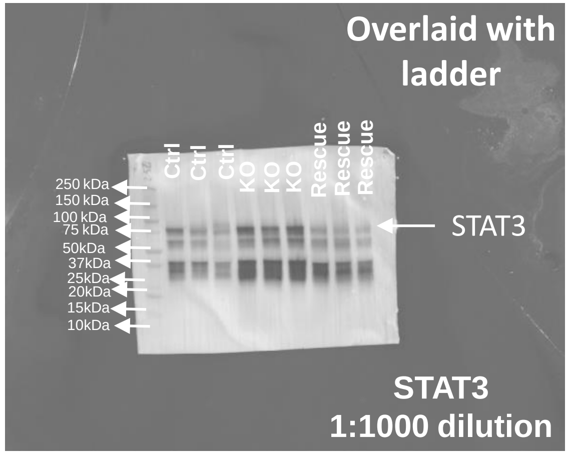

Western for STAT3

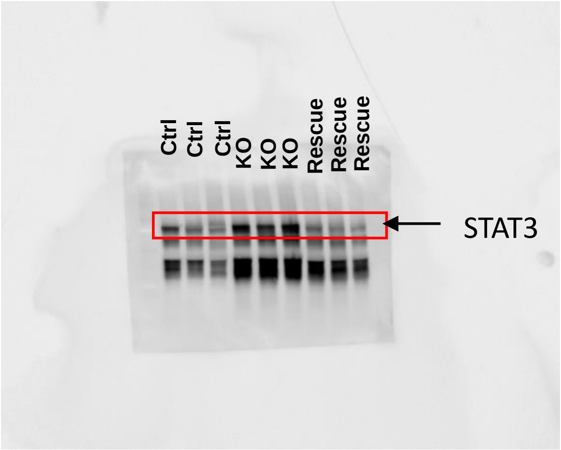

Same protein lysate was used for Blot239 [pSTAT3-Y705, SMARCB1 and beta-actin] and Blot252 [STAT3, SMARCB1 and beta-actin].

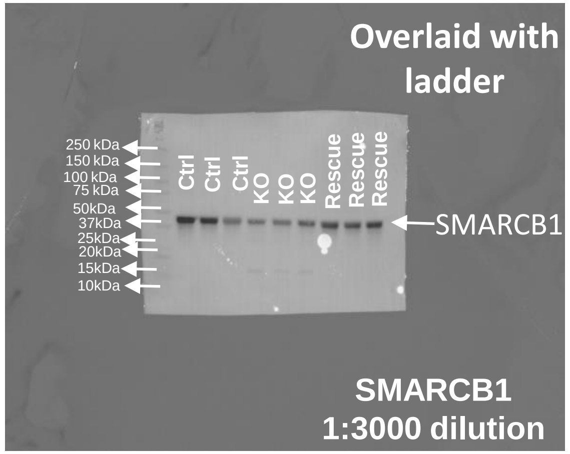

Western for SMARCB1

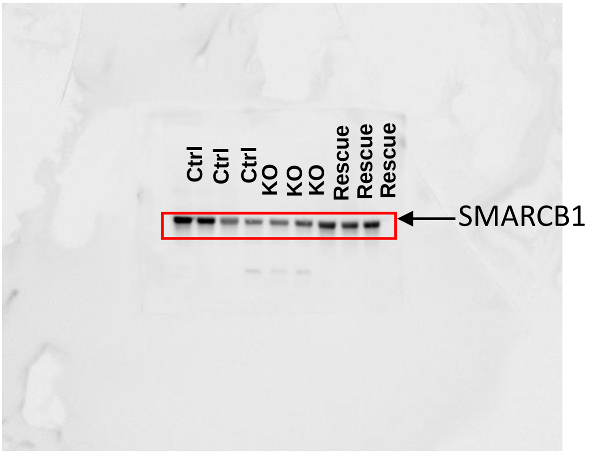

Area used for Figure 3D-Bottom panel

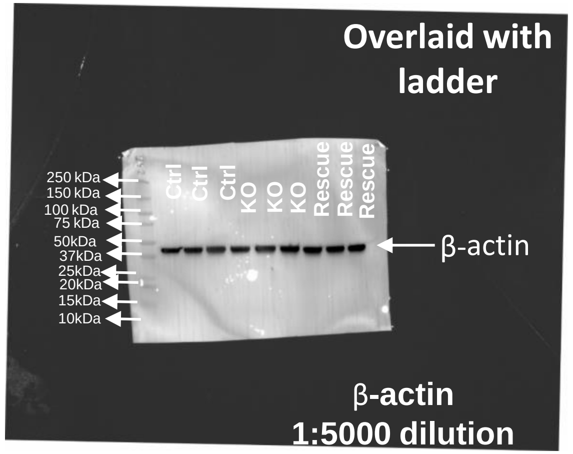

Western for β-actin

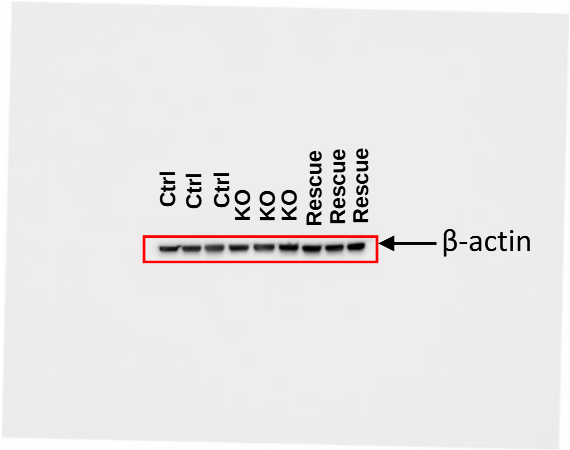

STAT3 1:1000
SMARCB1 1:3000
β-actin 1:5000

Blot 252

Raw image for Figure 3G

Western for pSTAT3 Y705

Raw image

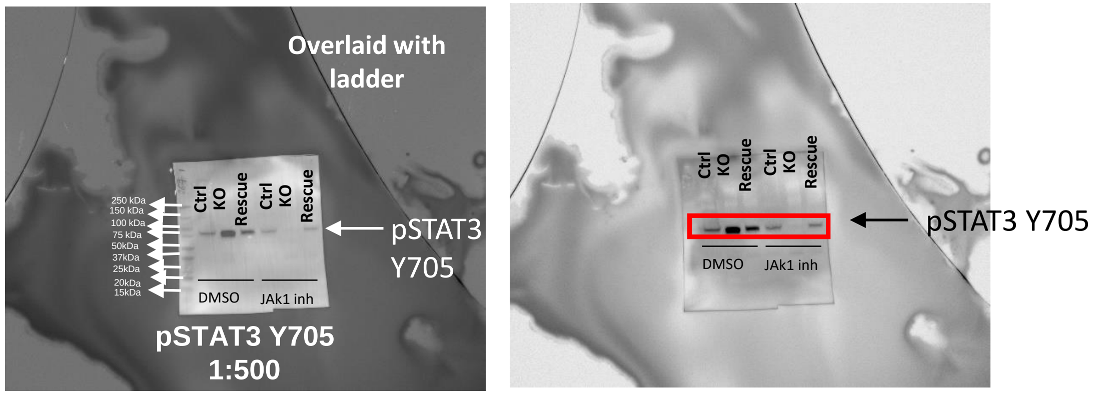

Western for SMARCB1

Raw image

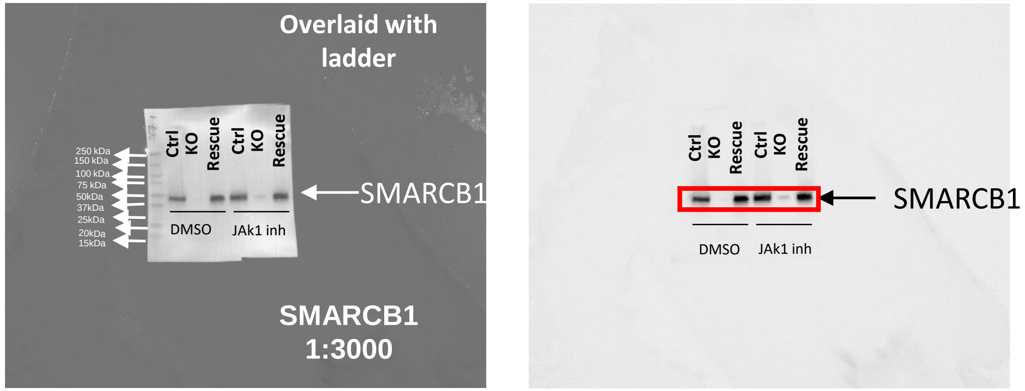

Western for STAT3

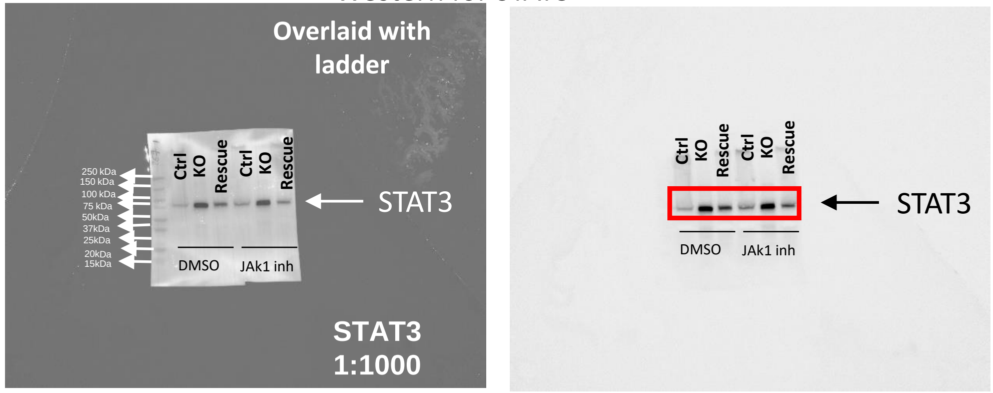

Western for beta-actin

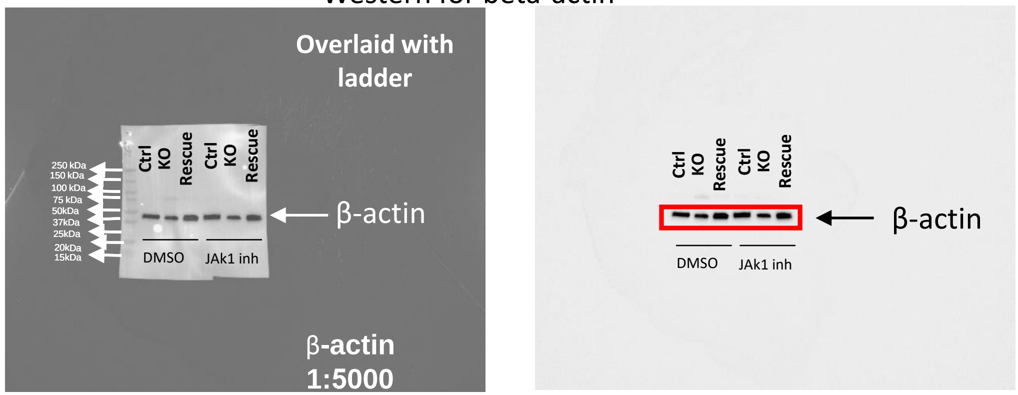

Area used for Figure 3G

pSTAT3 Y705 1:500
STAT3 1:1000
SMARCB1 1:3000
β-actin 1:5000

Blot267-1

Raw image for Figure 4A

Raw image

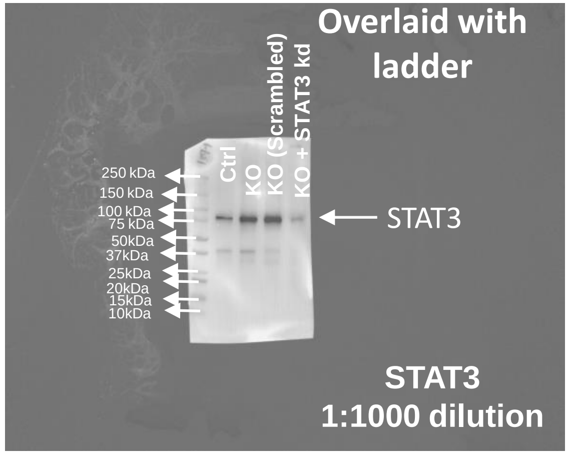

Western for STAT3

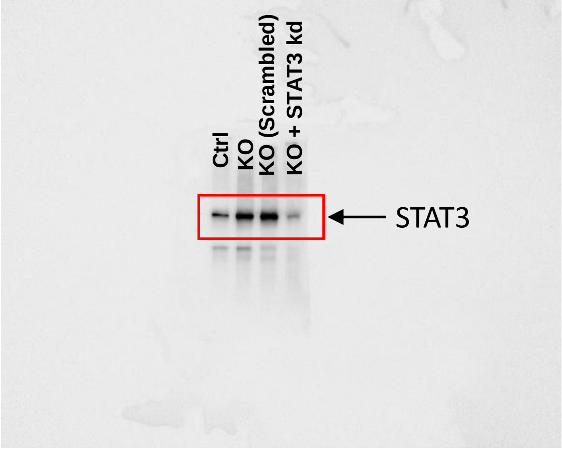

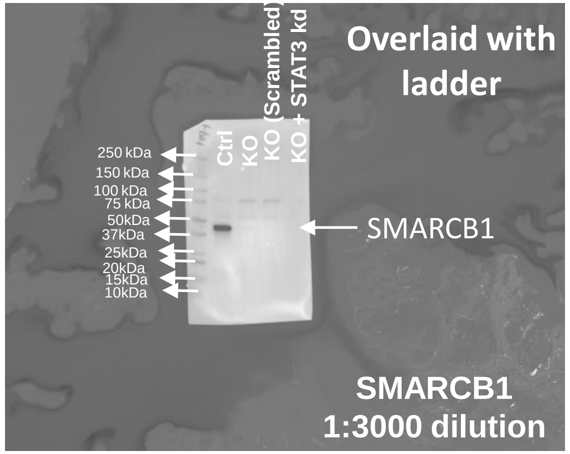

Western for SMARCB1

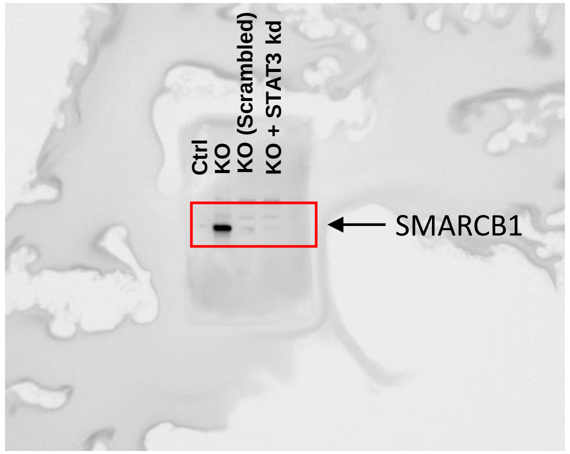

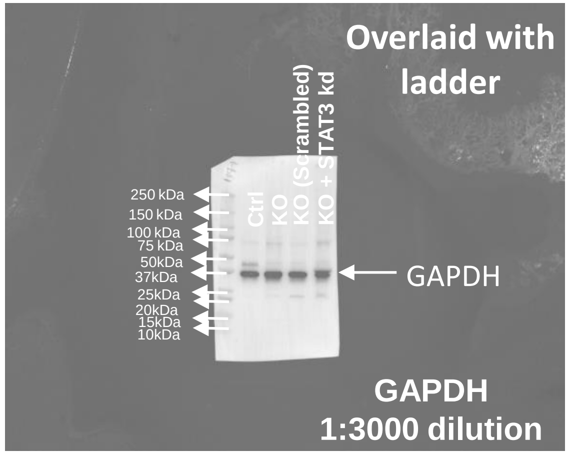

Western for GAPDH

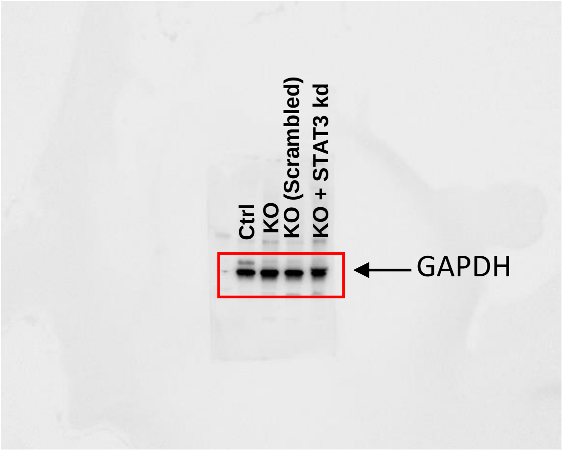

Area used for Figure 4A

STAT3 1:1000
SMARCB1 1:3000
GAPDH 1:3000

Blot 189-1

Raw image for Supplementary Figure 4B

Raw image

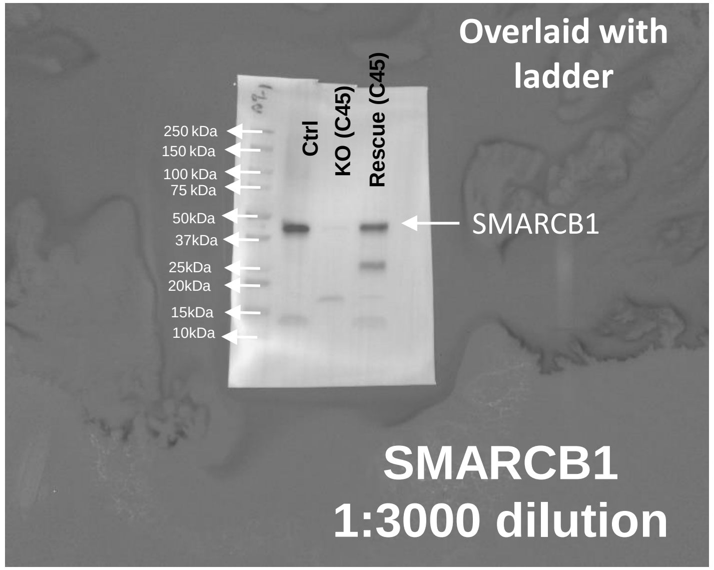

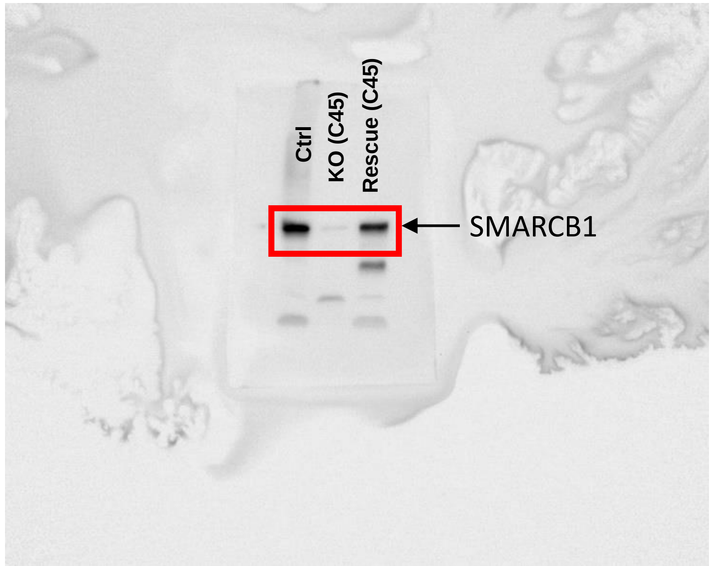

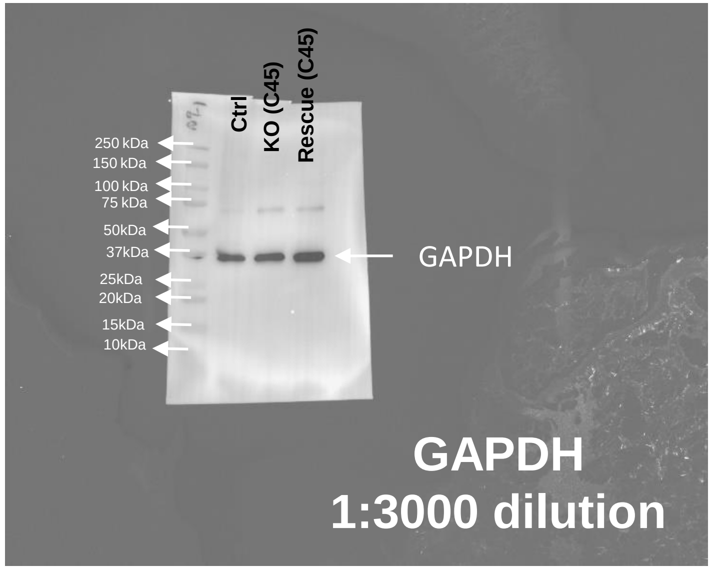

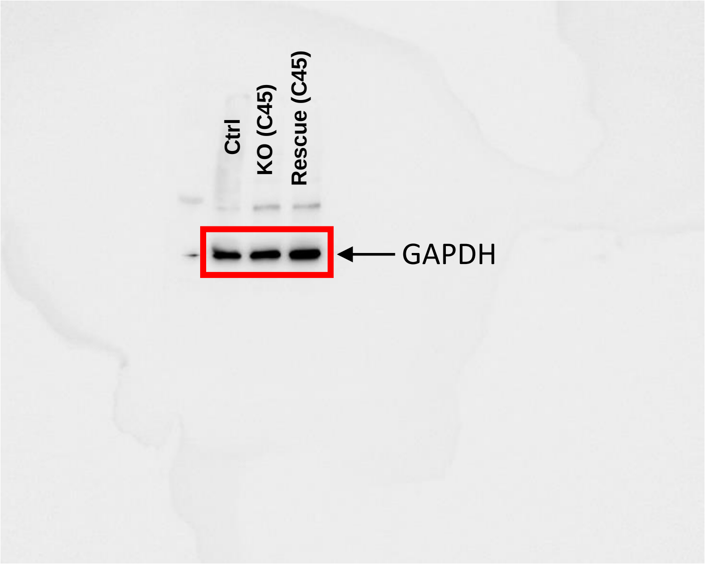

Area used for Supplementary Figure 4B

SMARCB1 1:3000
GAPDH 1:3000

Blot 109-1

Raw image for Supplementary Figure 4D

Western for SMARCA4

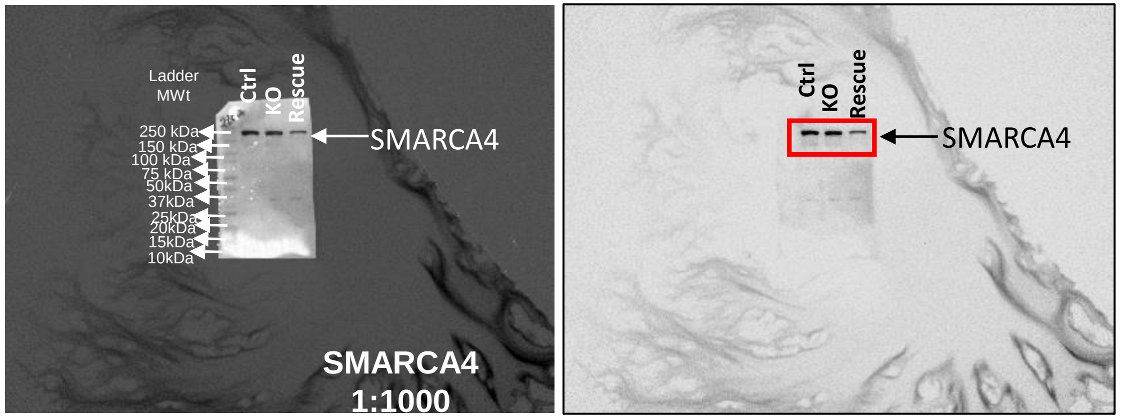

Western for SMARCB1

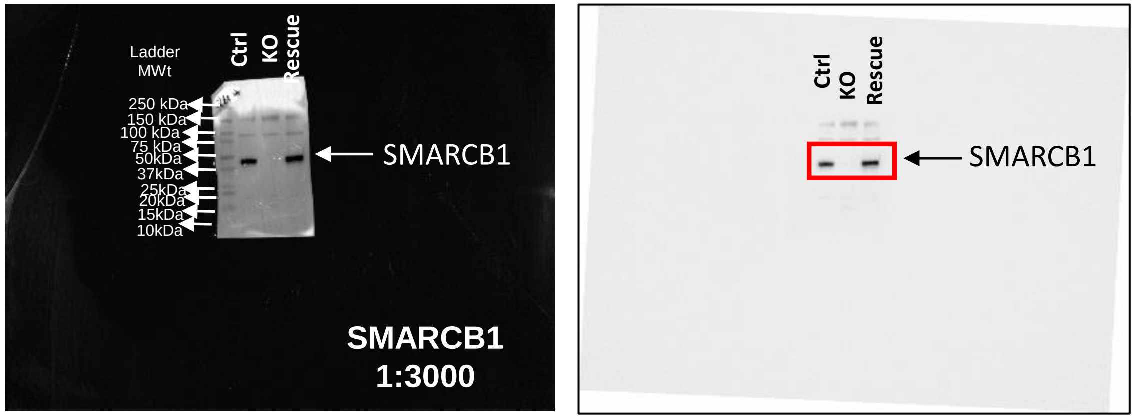

Western for SMARCC1

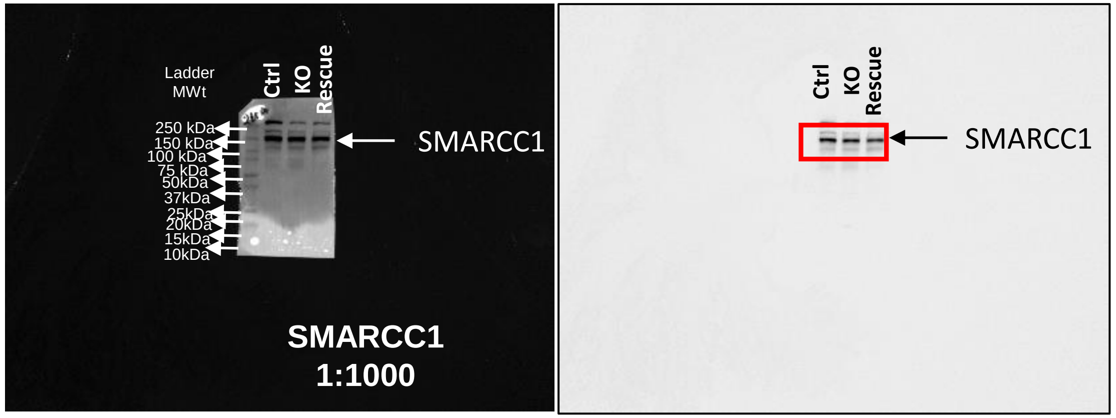

Western for GAPDH

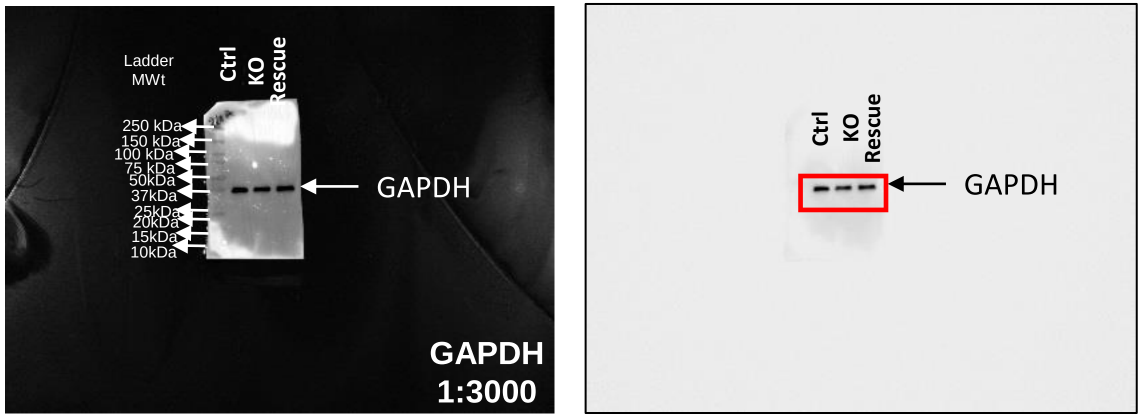

Western for SMARCC2

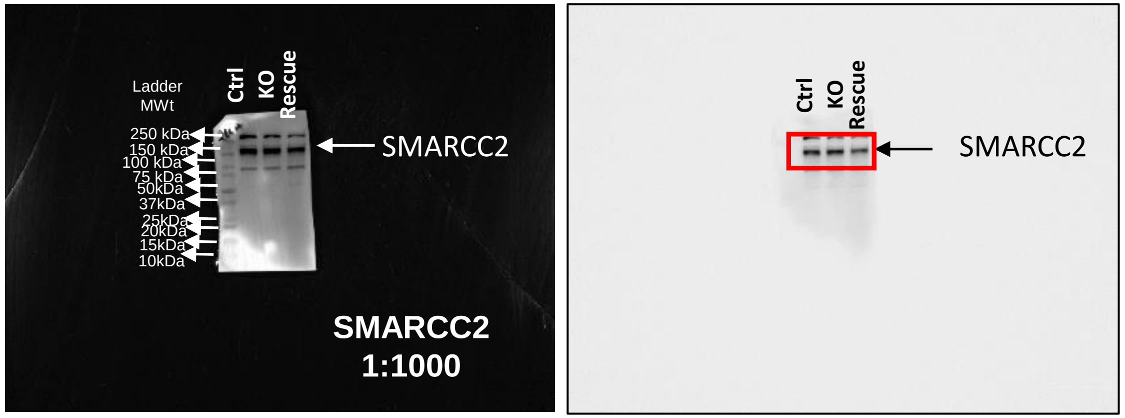

SMARCA4 1:1000
SMARCC1 1:1000
SMARCC2 1:1000
SMARCB1 1:3000
GAPDH 1:3000

Area used for Supplementary Figure 4D

Blot 260a

Raw image for Supplementary Figure 4F

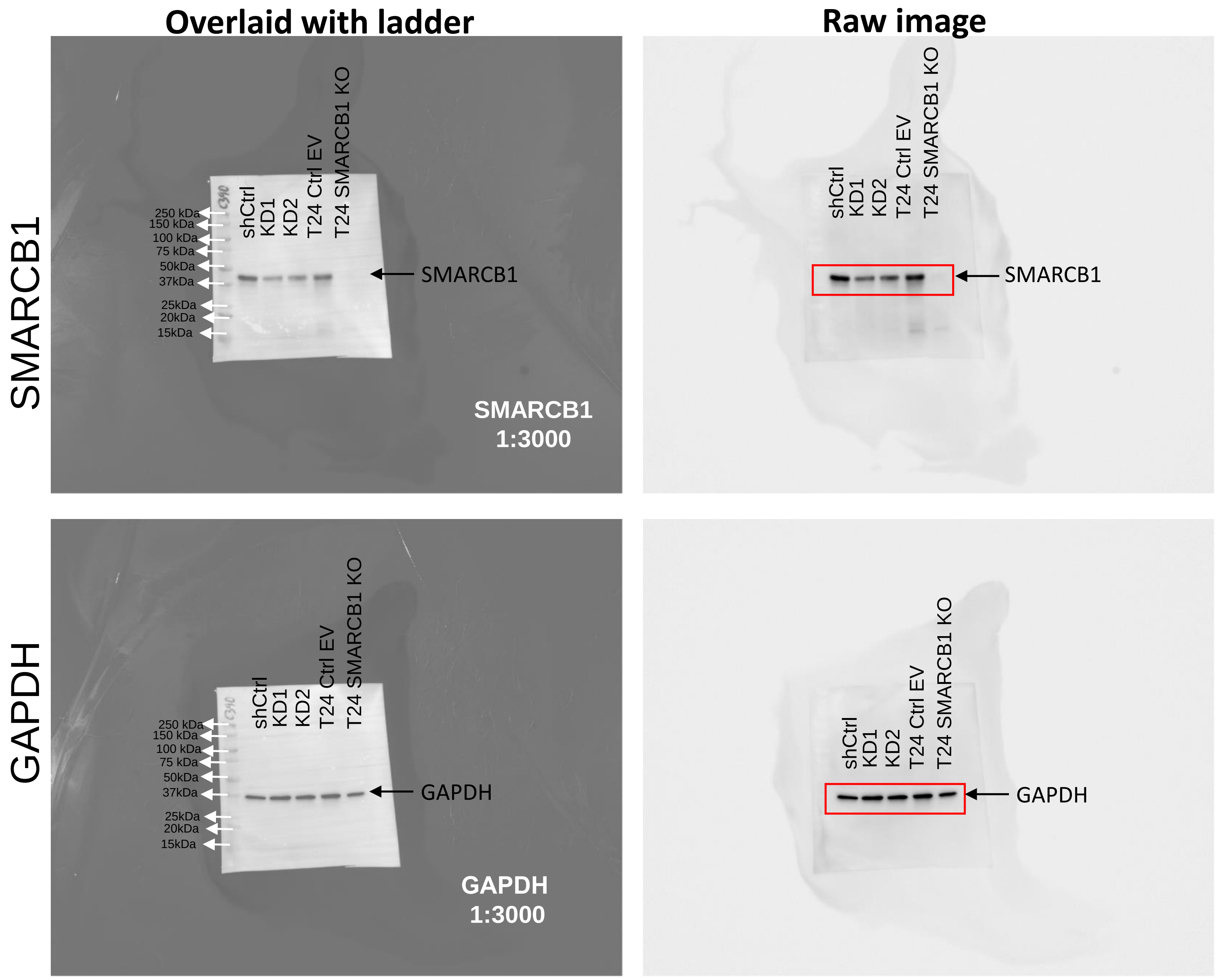

Area used for Supplementary Figure 4F

SMARCB1 – 1:3000 (CST)
GAPDH – 1:3000 (CST)

BlotC390

Raw image for Supplementary Figure 9C

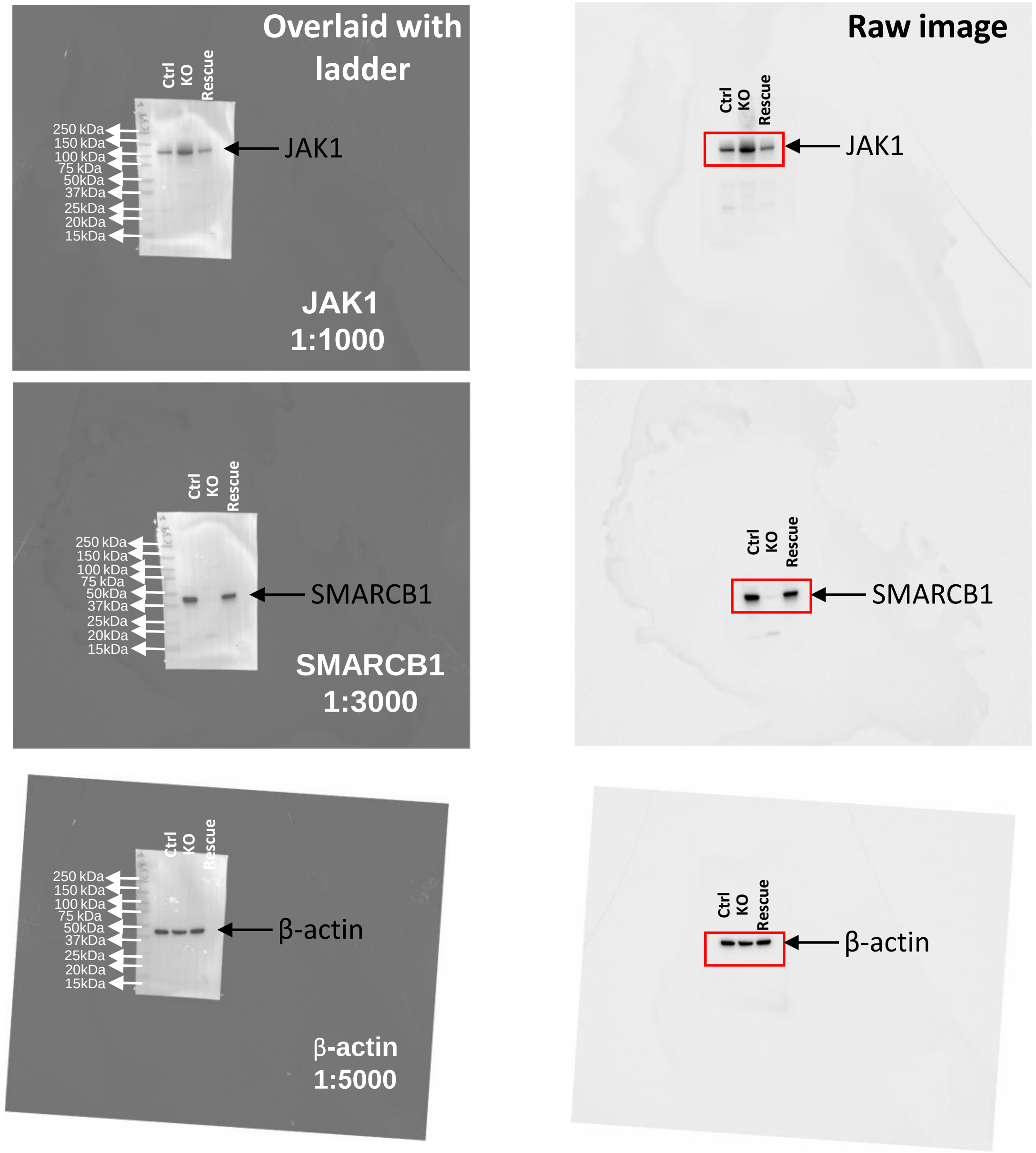

Western for JAK1

Western for SMARCB1

Western for β-actin

BlotC396-2

JAK1 1:1000
SMARCB1 1:3000
β-actin 1:5000

Area used for Supplementary Figure 9C

Raw image for Supplementary Figure 9G Top panel

Raw image

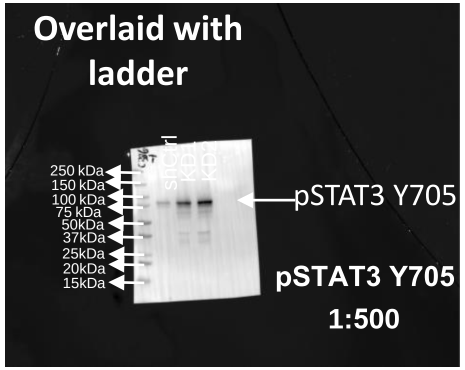

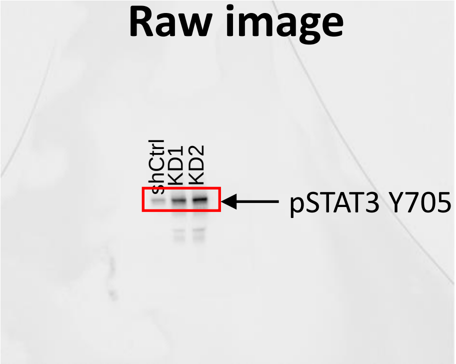

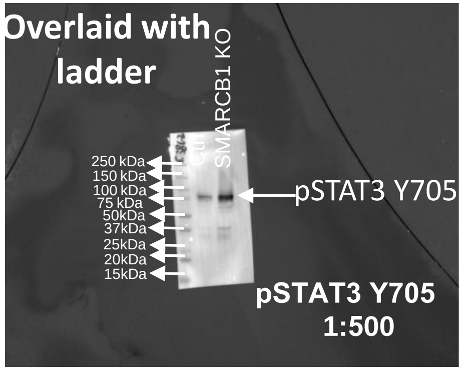

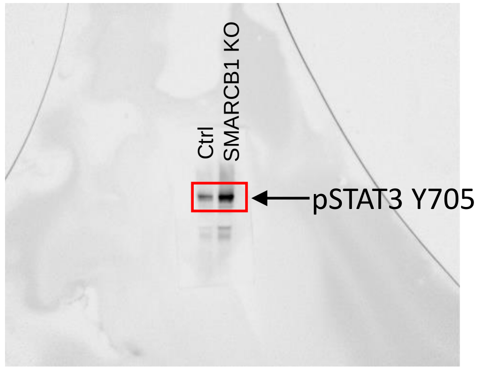

Western for pSTAT3-Y705

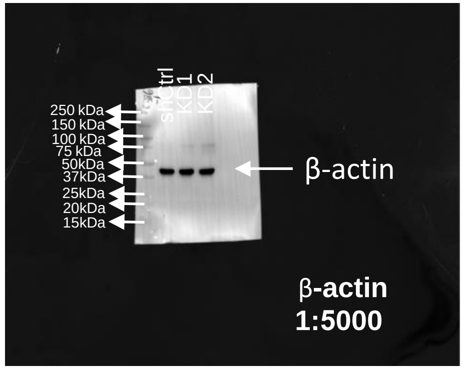

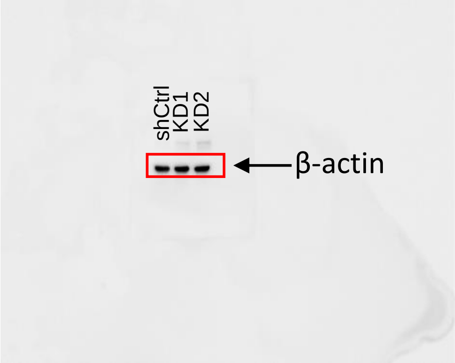

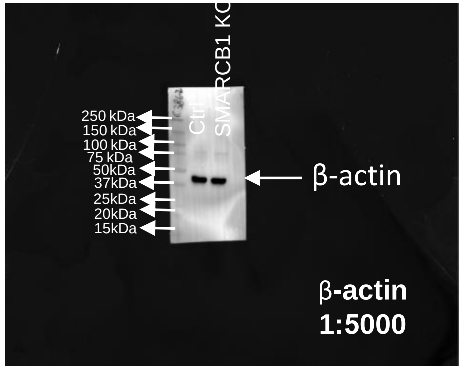

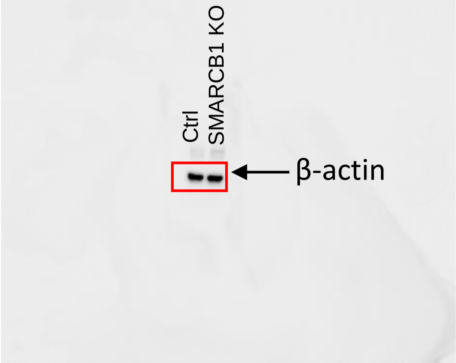

Western for β-actin

pSTAT3 Y705 1:500
β-actin 1:5000

Same protein lysates was used for BlotC386-1, BlotC386-2 [pSTAT3-Y705, and beta-actin] and BlotC385-1, BlotC385-2 [STAT3, and beta-actin].

BlotC386-1 and C386-2

Area used for Supplementary Figure 9G

Raw image for Supplementary Figure 9G Bottom panel

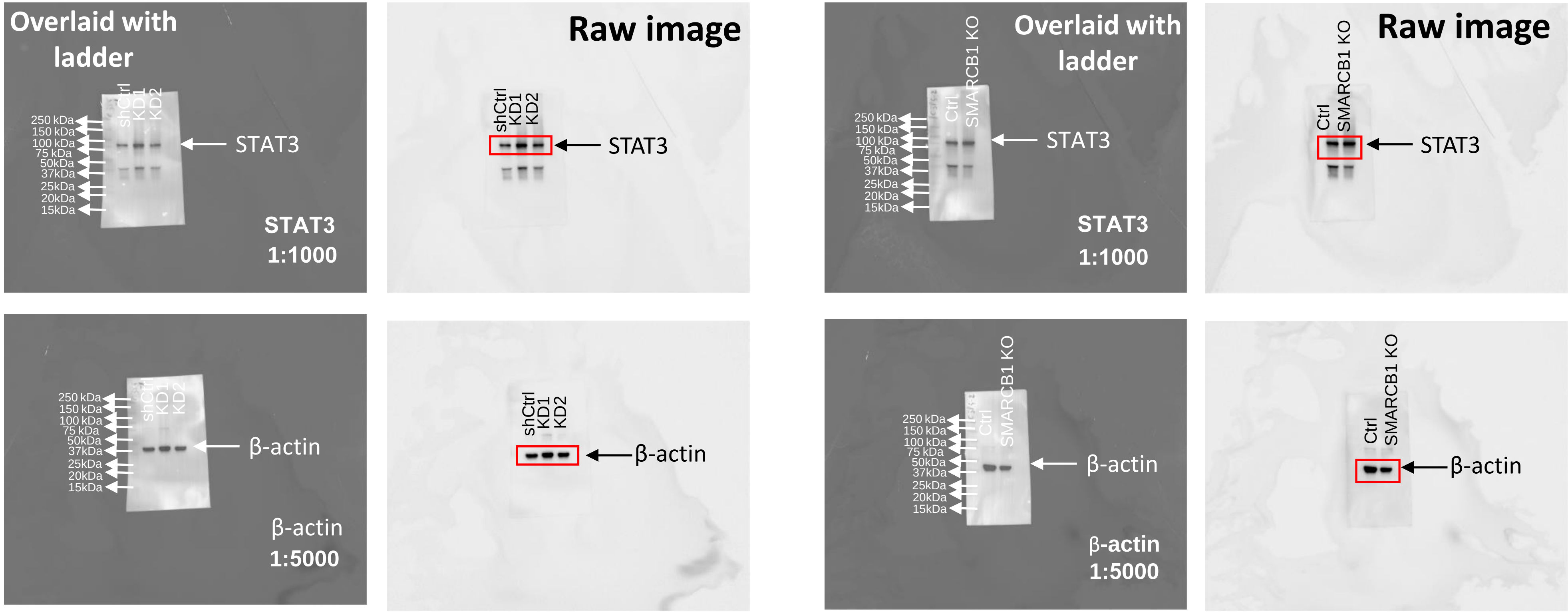

Western for STAT3

Western for β-actin

STAT3 1:1000
β-actin 1:5000

Same protein lysates was used for BlotC386-1, BlotC386-2 [pSTAT3-Y705 and beta-actin] and BlotC385-1, BlotC385-2 [STAT3 and beta-actin].

BlotC385-1 and C385-2

Area used for Supplementary Figure 9G

Raw image for Supplementary Figure 11A

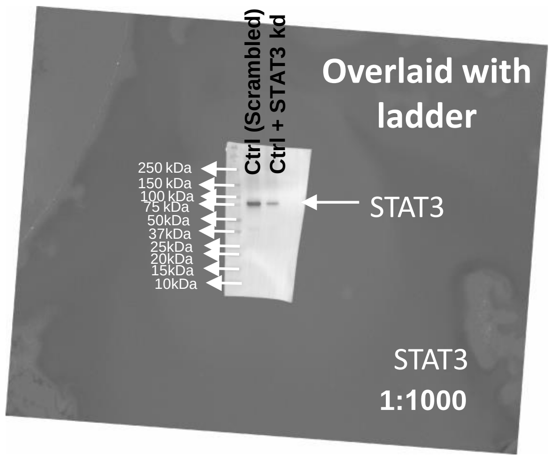

Western for STAT3

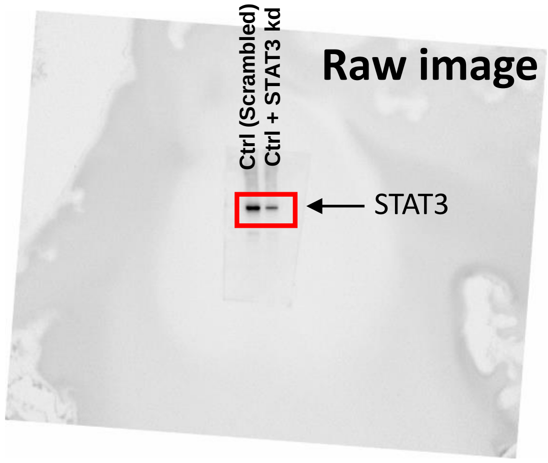

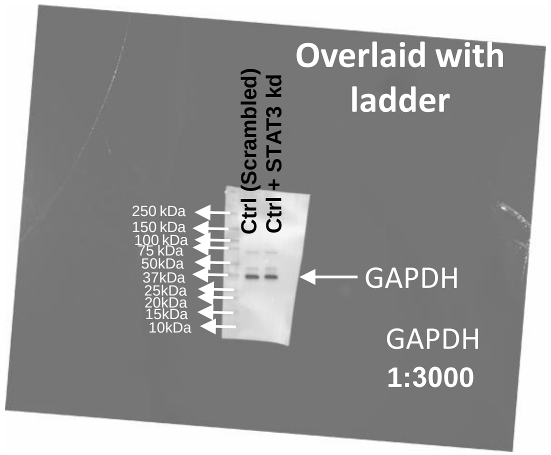

Western for GAPDH

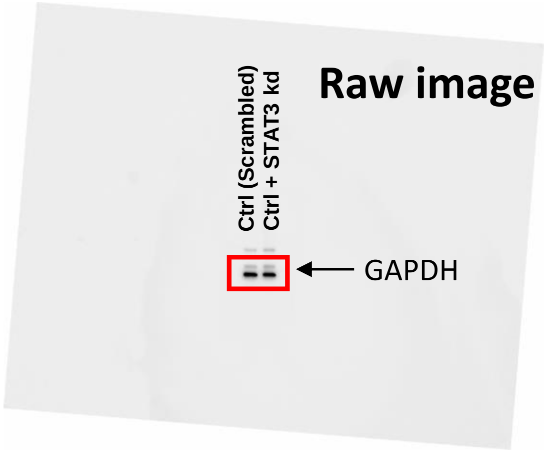

Area used for Supplementary Figure 11A

STAT3 1:1000
GAPDH 1:3000

Blot 191-2

Raw images associated with Supplementary Figure 13B

SMARCB1 staining

BL8091

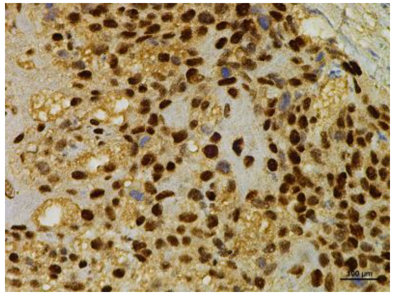

Staining Score

Score 3
Positive cells 90%

TM00020

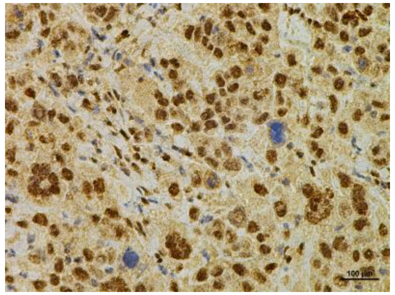

Staining Score

Score 2
Positive cells 80%

BL8091

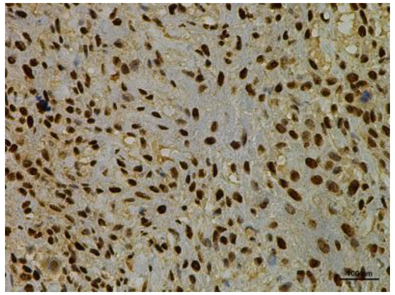

Score 3
Positive cells 90%

TM00020

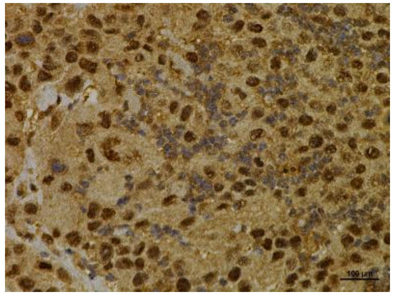

Score 2
Positive cells 80%

IRS score was used for Supplementary Figure 13B bottom panel

BL8091

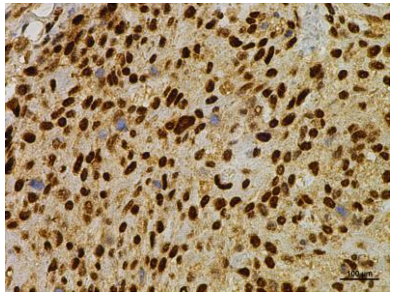

Score 3
Positive cells 90%

TM00020

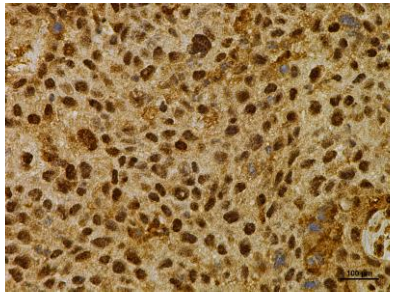

Score 2
Positive cells 80%

Area used for representation for Supplementary Figure 13B

Positive and negative controls associated with Supplementary Figure 13B

SMARCB1 staining

Negative control

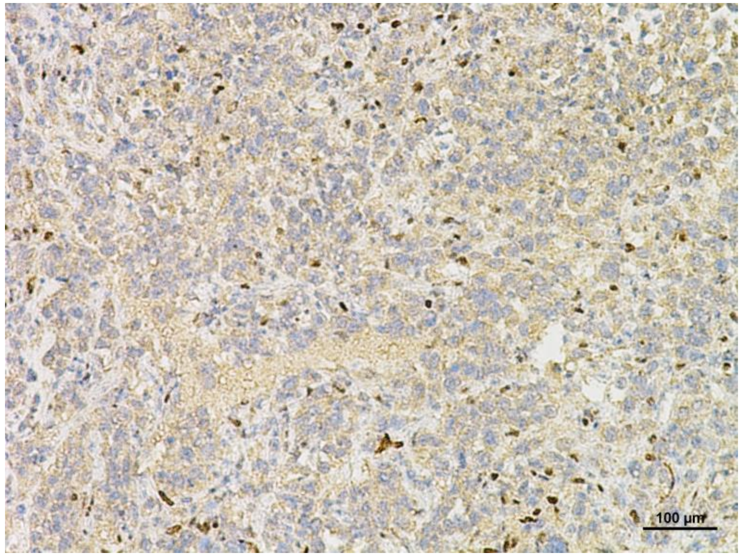

Positive control

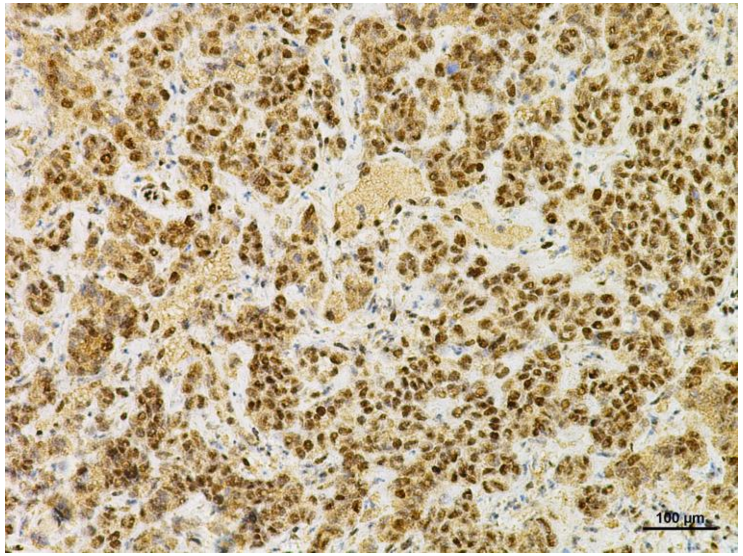

Raw images associated with Supplementary Figure 13C

pSTAT3 Y705 staining

BL8091

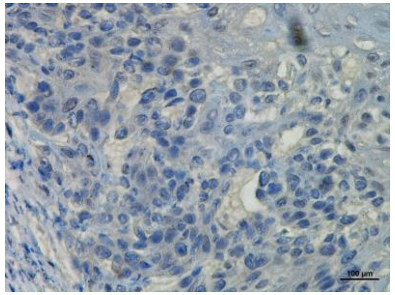

Staining Score

Score 0
Positive cells 0%

TM00020

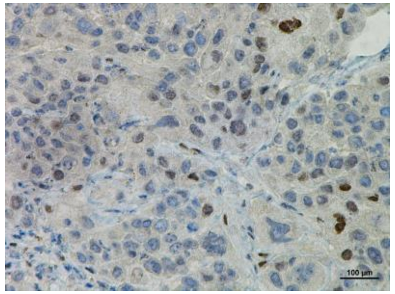

Staining Score

Score 2
Positive cells 10%

BL8091

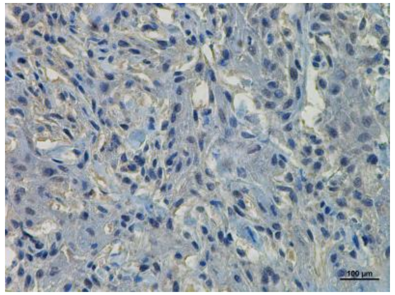

Score 0
Positive cells 0%

TM00020

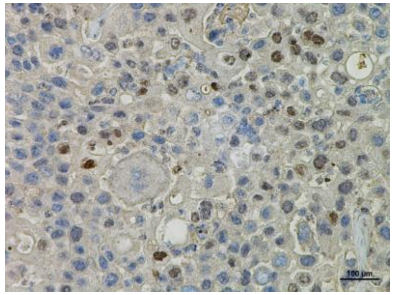

Score 2
Positive cells 10%

IRS score was used for Supplementary Figure 13C bottom panel

BL8091

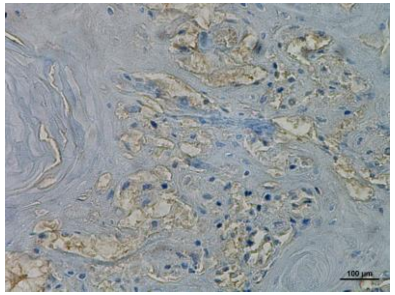

Score 0
Positive cells 0%

TM00020

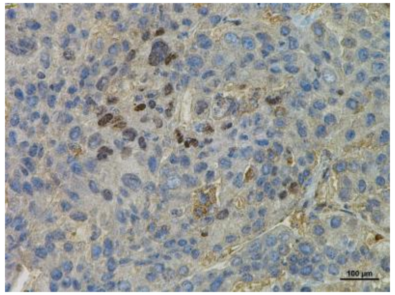

Score 2
Positive cells 5%

Area used for representation for Supplementary Figure 13C

Positive and negative controls associated with Supplementary Figure 13C

pSTAT3 Y705 staining

Negative control

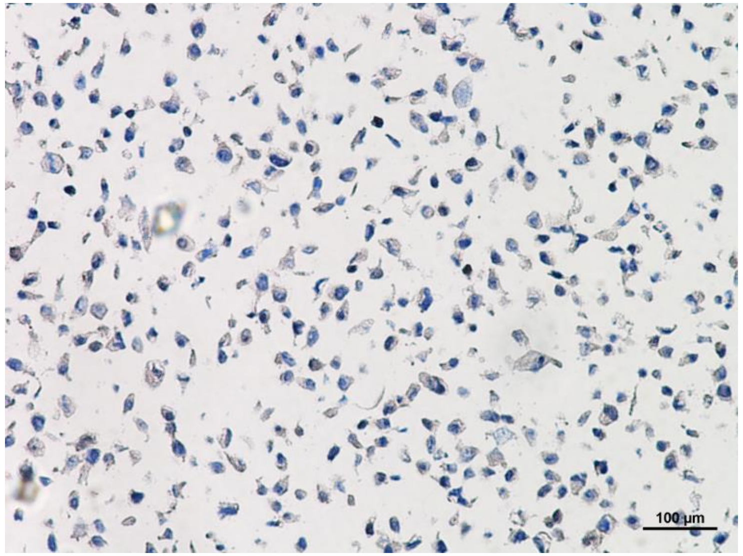

Positive control

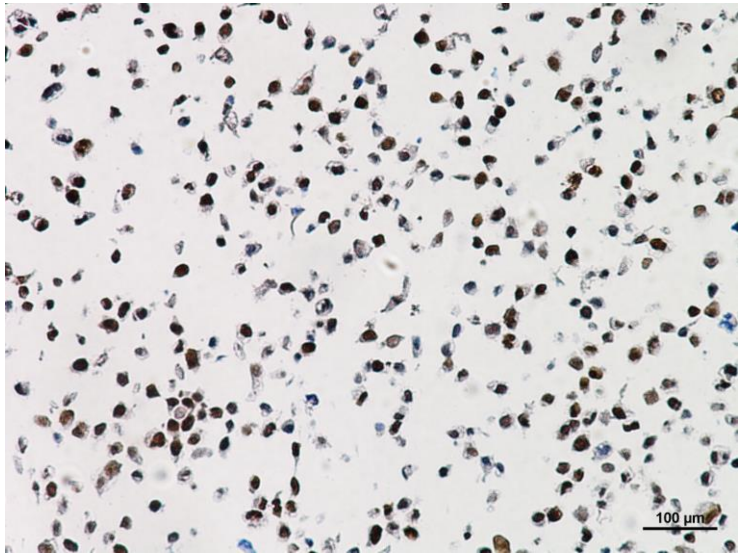

Supplement: Supplementary file 15 — Source Data [file 41467_2024_45132_MOESM15_ESM.zip › 41467_2024_45132_MOESM15_ESM/Source Data/Source data associated with Figures for Westerns and IHC.pdf]
