# Supplementary material for: The IL6/JAK/STAT3 signaling axis is a therapeutic vulnerability in SMARCB1-deficient bladder cancer
Source: Nat Commun. 2024 Feb 14;15:1373. doi: 10.1038/s41467-024-45132-2 (PMC10867091; doi:10.1038/s41467-024-45132-2)

Source data-01 associated with
Figure 1A

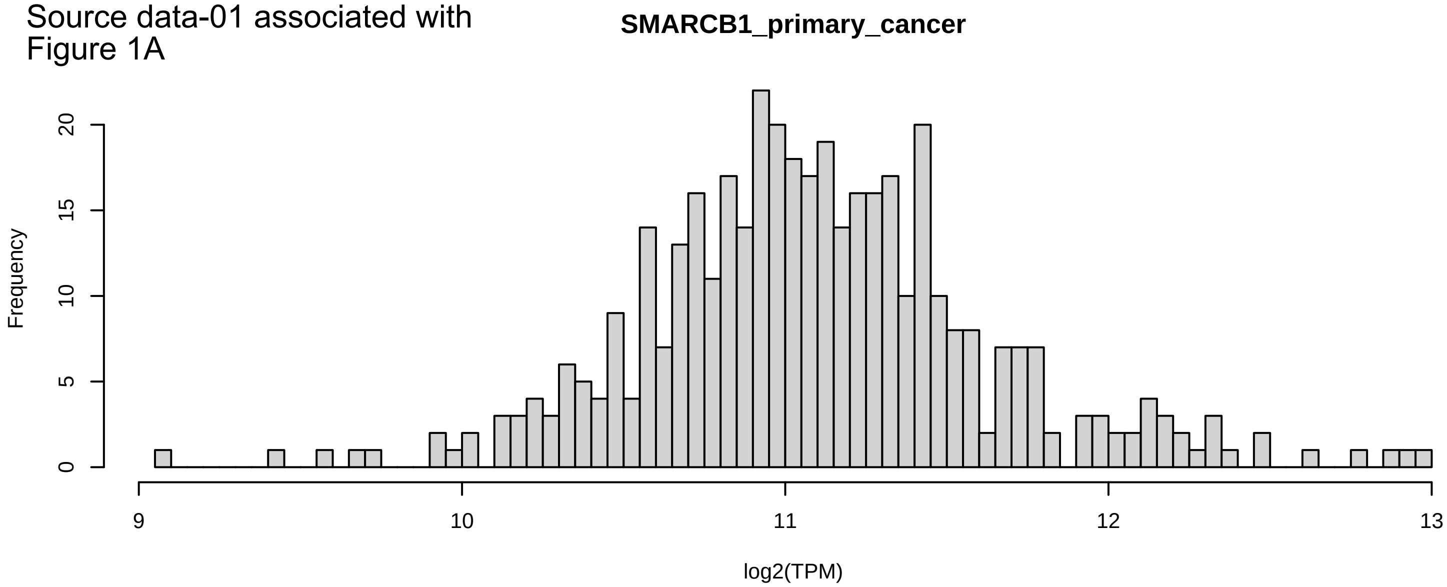

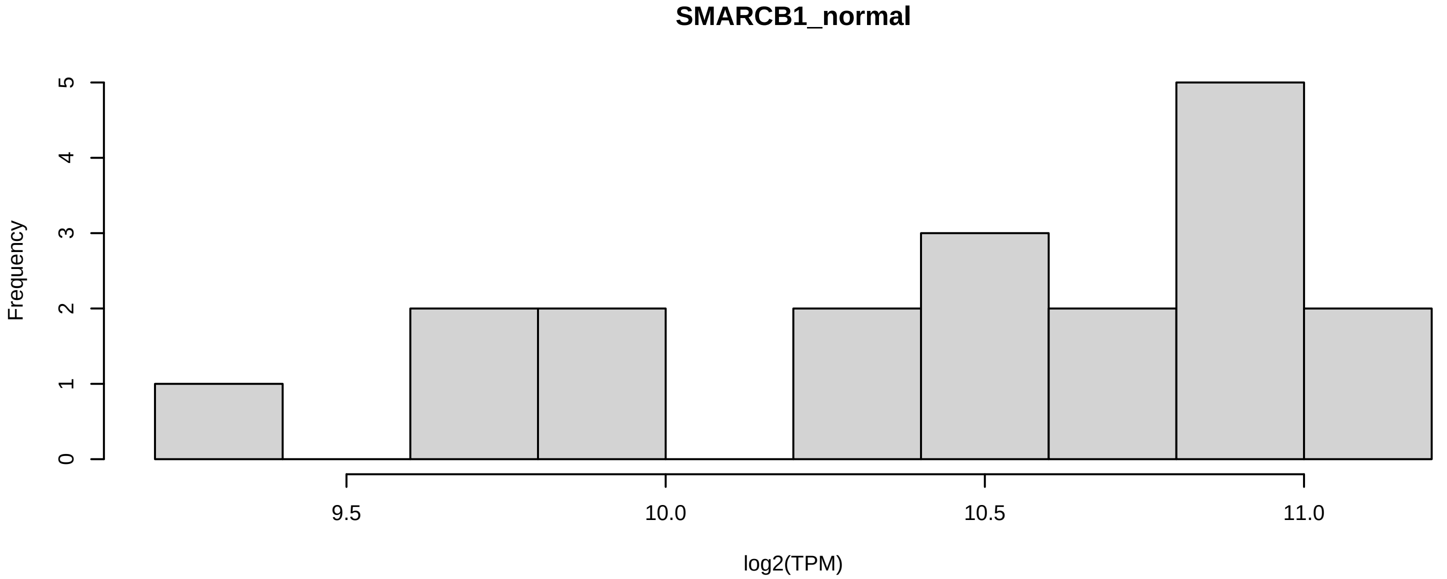

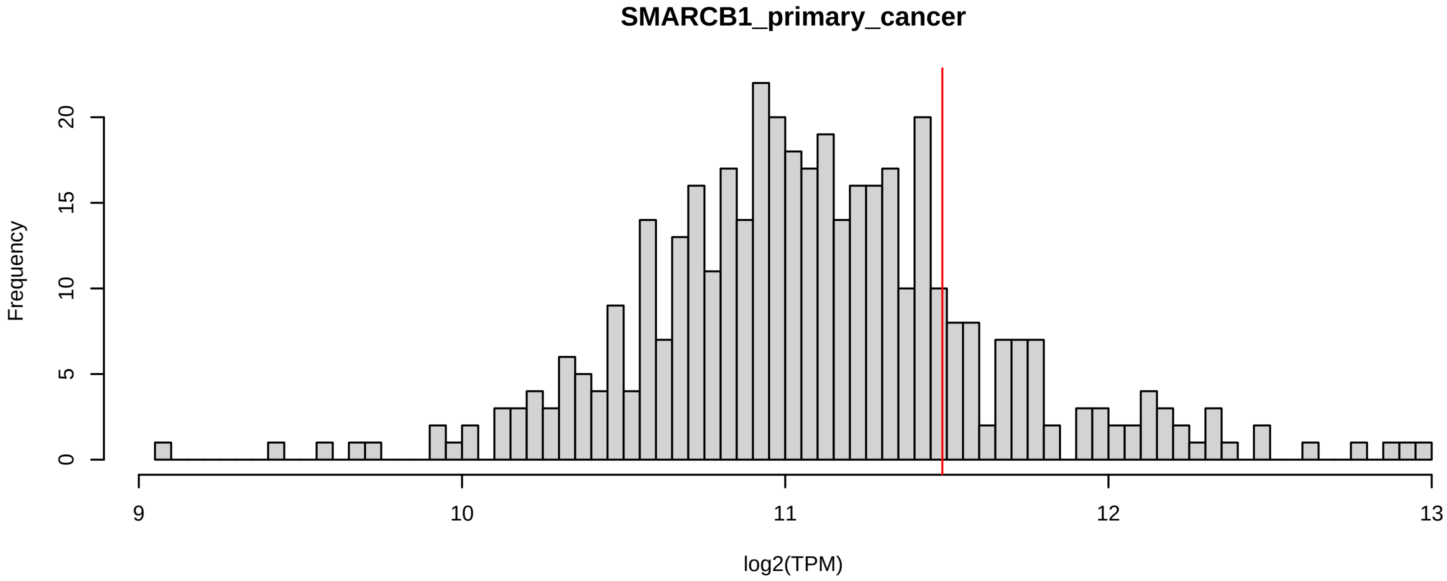

Supplement: Supplementary file 15 — Source Data [file 41467_2024_45132_MOESM15_ESM.zip › 41467_2024_45132_MOESM15_ESM/Source Data/Source data-01 Associated with Fig.1A.pdf]
